# Supplementary figures and images for: Time dependent genetic analysis links field and controlled environment phenotypes in the model C4 grass Setaria
Source: PLoS Genet. 2017 Jun 23;13(6):e1006841. doi: 10.1371/journal.pgen.1006841 (PMC5507400; doi:10.1371/journal.pgen.1006841)

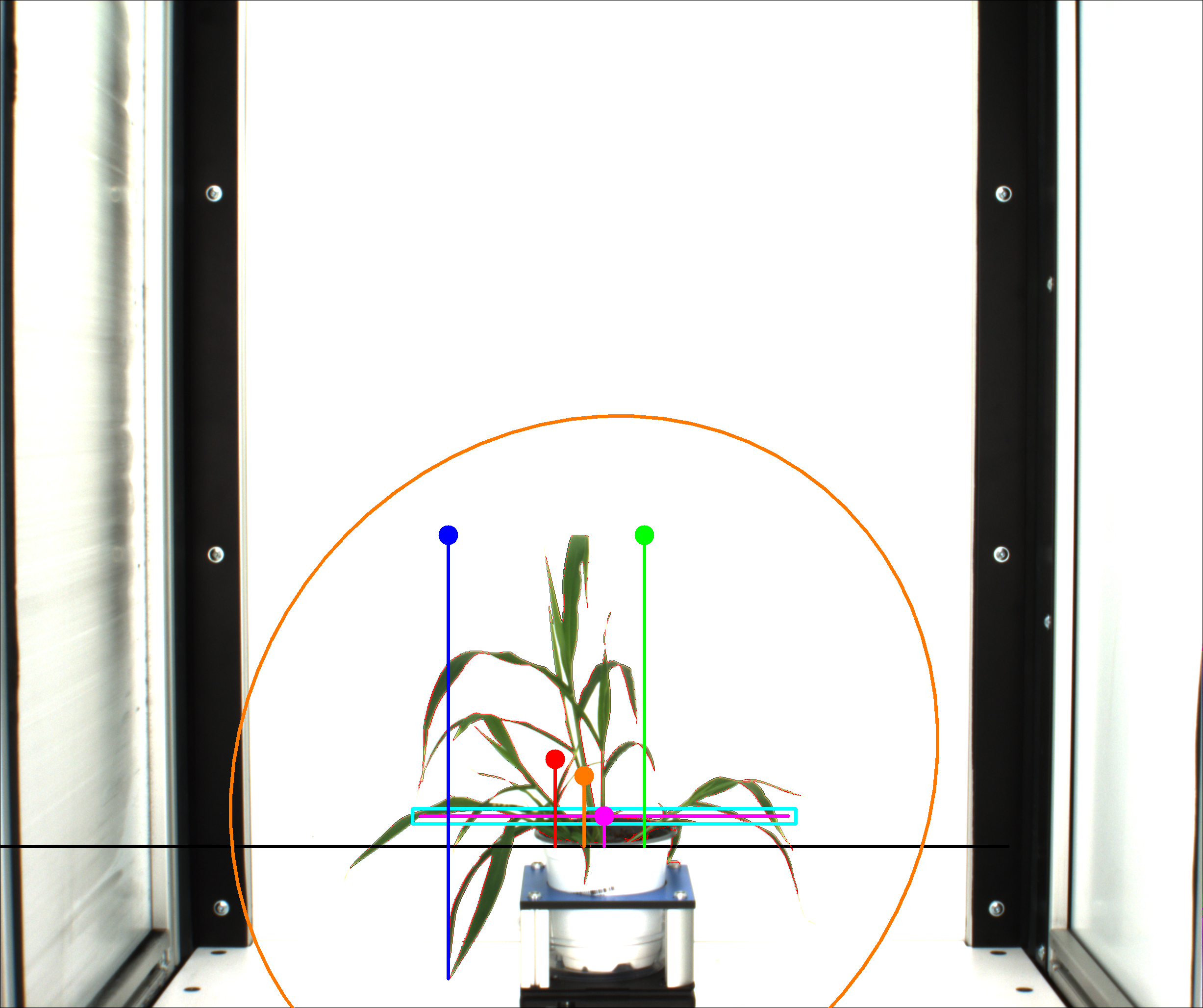

Supplement: S1 Fig — Extent_y metric is in blue, height_above_bound is in green, centroid_y is in red, elipse_center_y is in orange and canopy_height is illustrated in purple. (PNG) [file pgen.1006841.s001.png]

## H2 (mean)

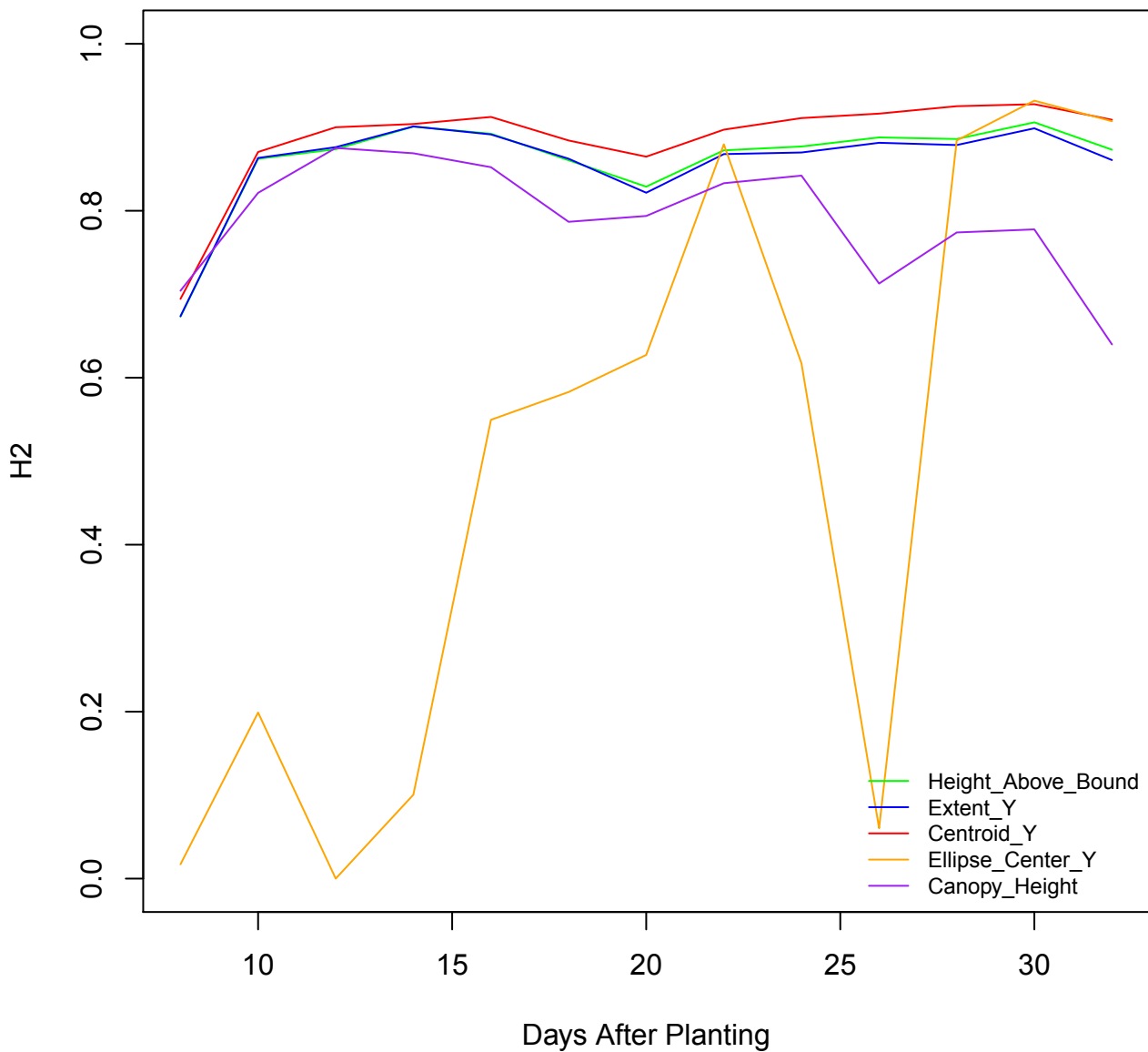

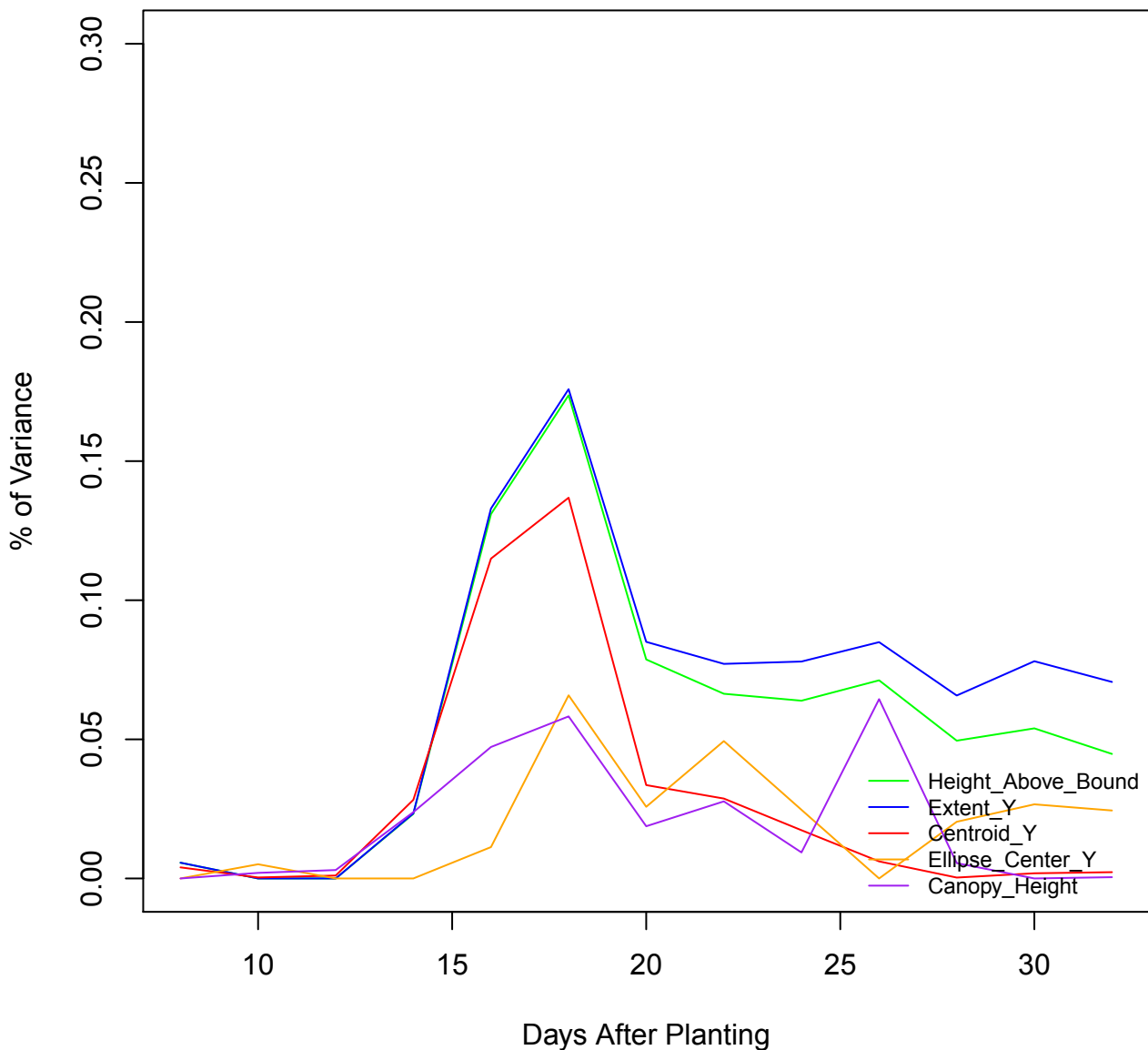

## G X Treatment (mean)

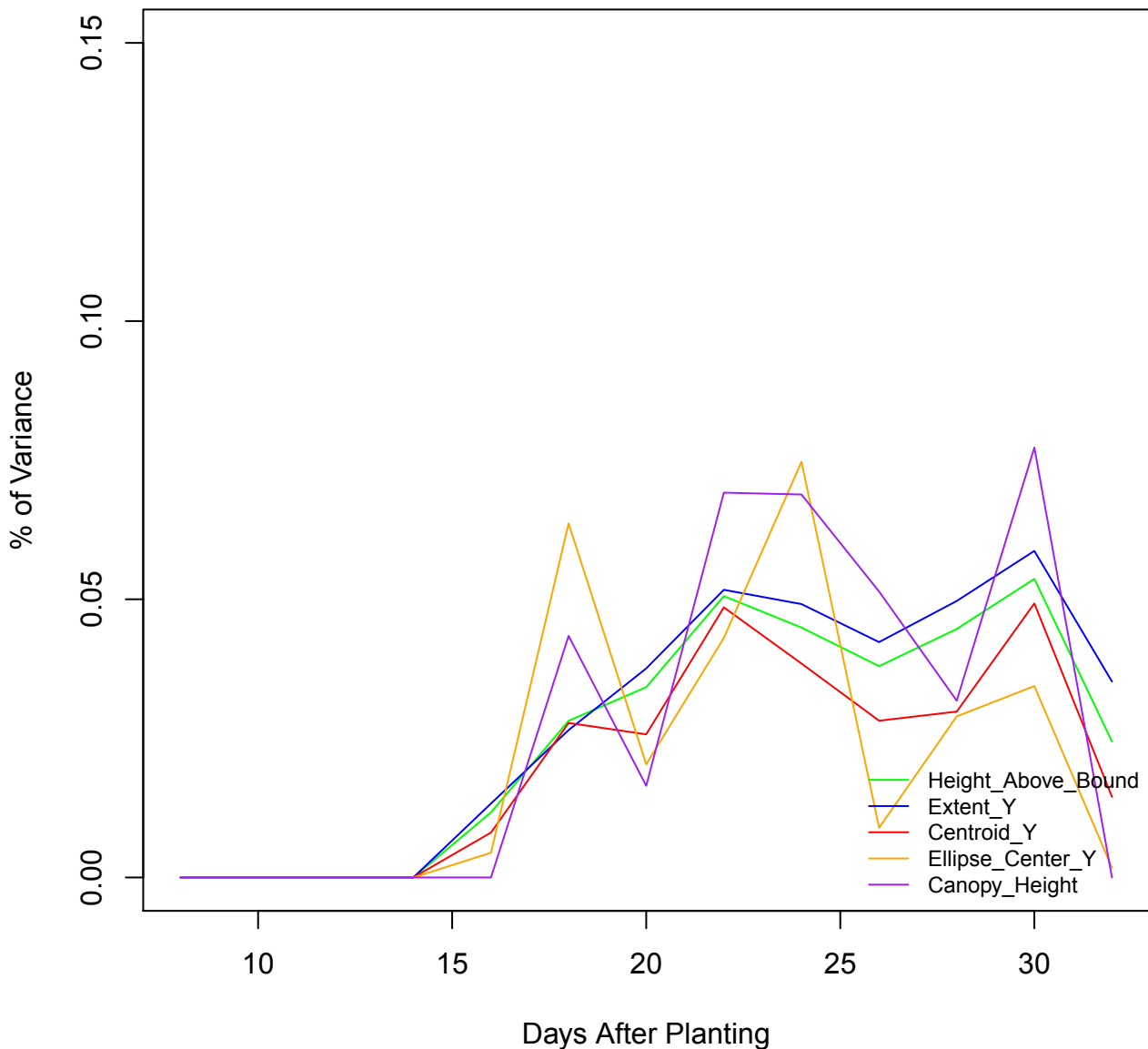

Supplement: S2 Fig — A) The broadsense heritability of ‘height_above_bound’, ‘Centroid_Y’, ‘Extent_Y’, ‘Ellipse_Center_Y’, and canopy height plotted across all time points in the Bellweather experiment. B) The proportion of variance attributed to treatment as measured using ‘height_above_bound’, ‘Centroid_Y’, ‘Extent_Y’, ‘Ellipse_Center_Y’, and canopy height metrics throughout all time points in the Bellweather experiment. C) The proportion of variance attributed to genotype x treatment partition as measured using ‘height_above_bound’, ‘Centroid_Y’, ‘Extent_Y’, ‘Ellipse_Center_Y’, and canopy height metrics throughout all time points in the Bellweather experiment. (PDF) [file pgen.1006841.s002.pdf]

## height\_above\_bound

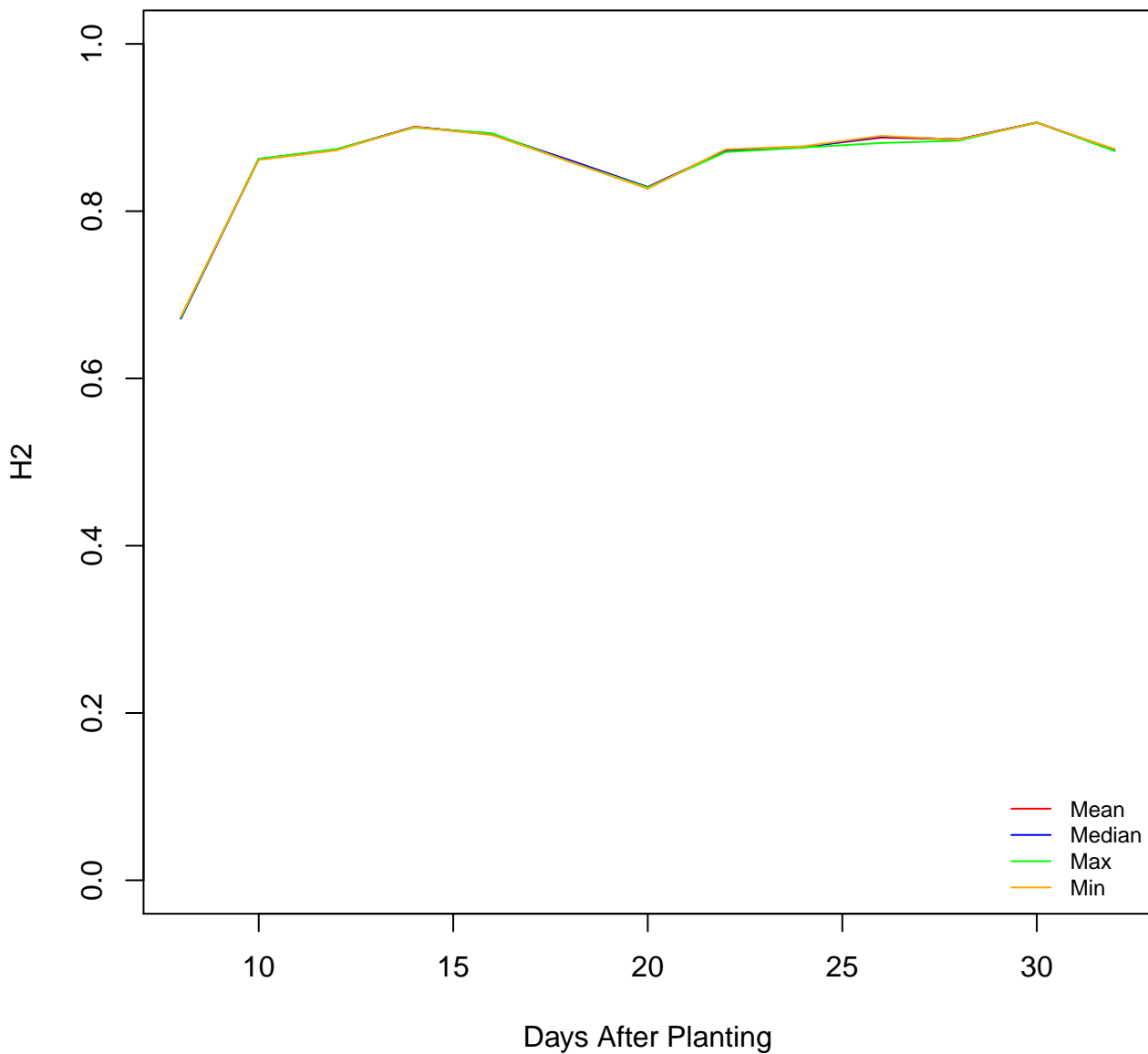

Supplement: S3 Fig — The broad sense heritability of ‘height_above_bound’ is roughly equivalent given the 4 types of summarization functions is used (mean, median, max, min). (PDF) [file pgen.1006841.s003.pdf]

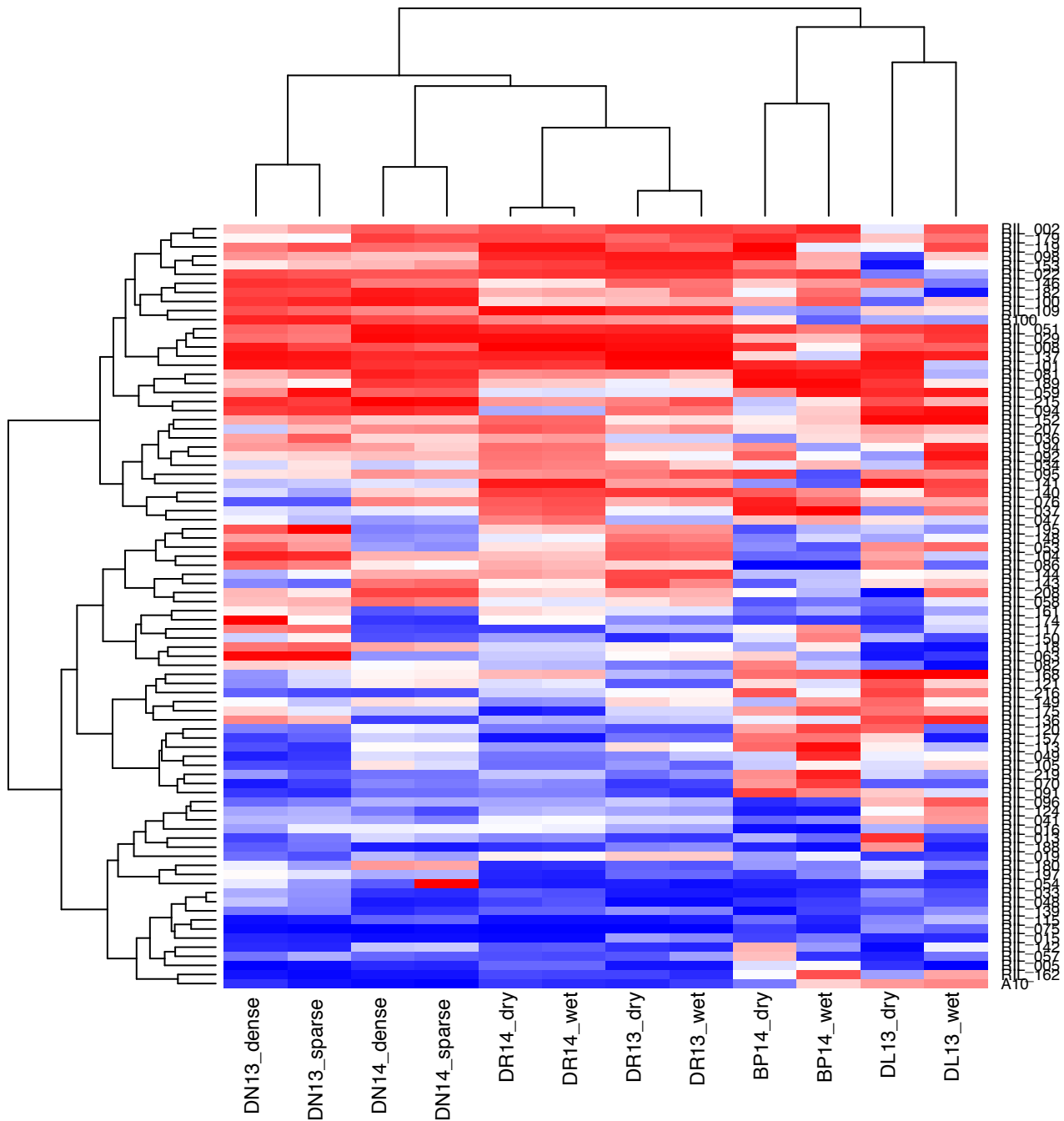

Supplement: S4 Fig — (PDF) [file pgen.1006841.s004.pdf]

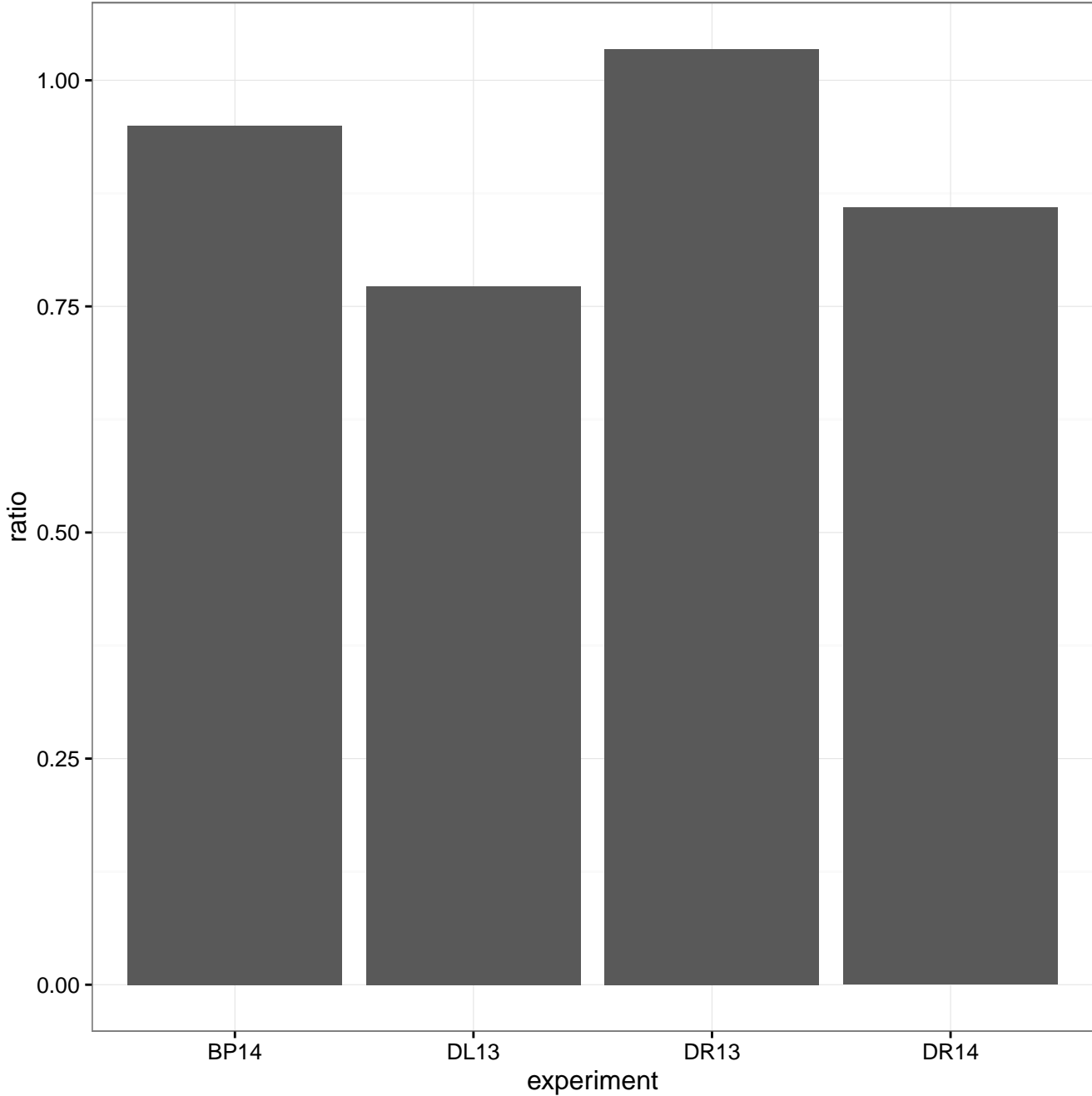

Supplement: S5 Fig — (PDF) [file pgen.1006841.s005.pdf]

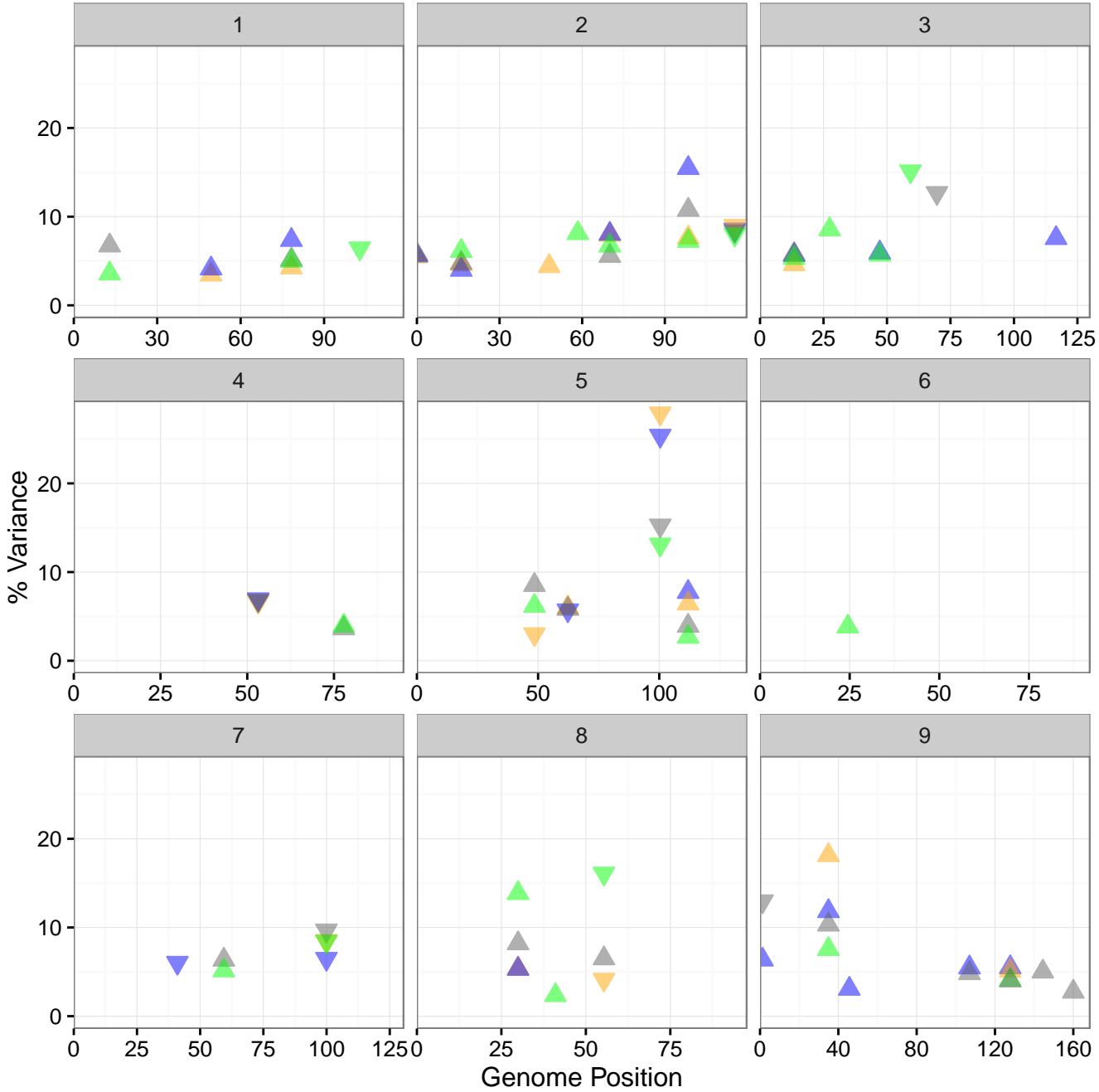

Supplement: S6 Fig — Each box corresponds to an individual chromosome, where the values along the x-axis are chromosome position (centimorgan) and values along the y-axis denote the proportion of genetic variance explained by the QTL. Each triangle represents a single QTL detected, where the color indicates the treatment condition the QTL was identified in (blue represents wet, orange corresponds to dry, green indicates sparse planting density whereas grey denotes dense planting.) and direction of the arrow corresponds the directional effect of the B100 parental allele. (PDF) [file pgen.1006841.s006.pdf]

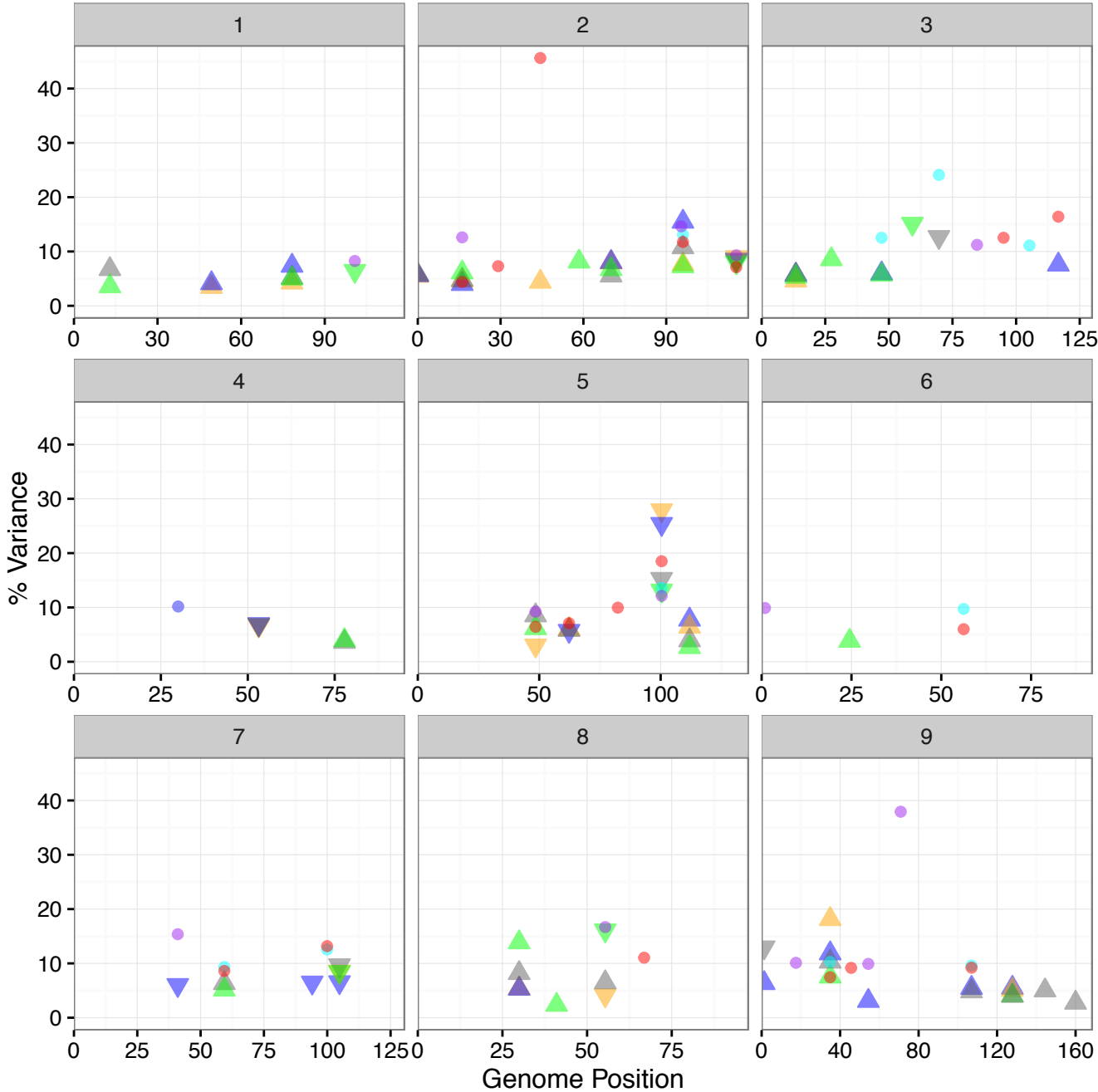

Supplement: S8 Fig — The location of trait difference QTLs is plotted as circles colored by their difference type. QTLs detected by mapping on the numerical difference between treatment blocks are plotted in cyan. QTLs detected by mapping on the relative difference between treatment blocks are plotted in purple. QTLs detected by mapping on the ratio between treatment blocks are plotted in red. (PDF) [file pgen.1006841.s008.pdf]

# DN13

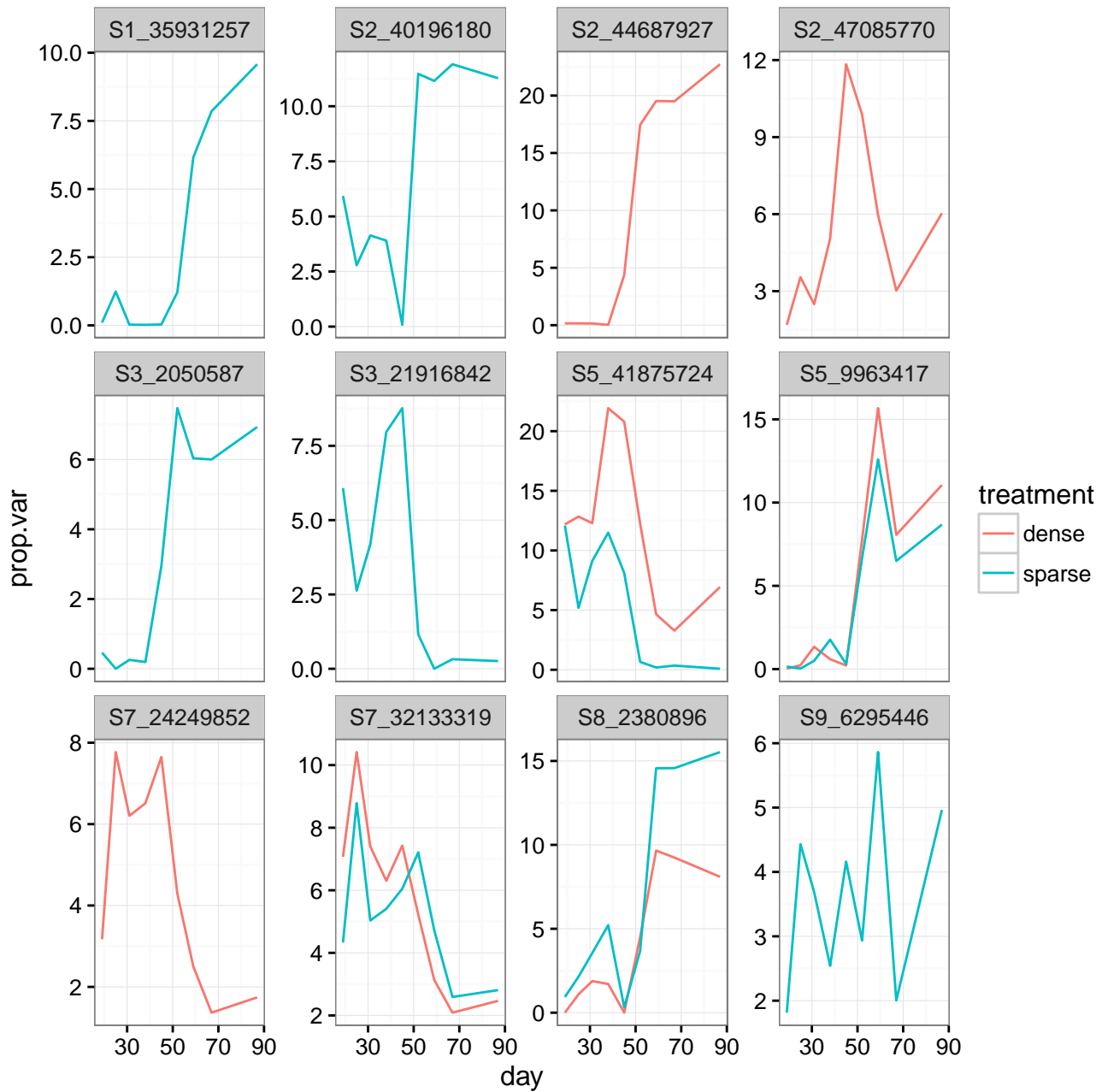

# DN14

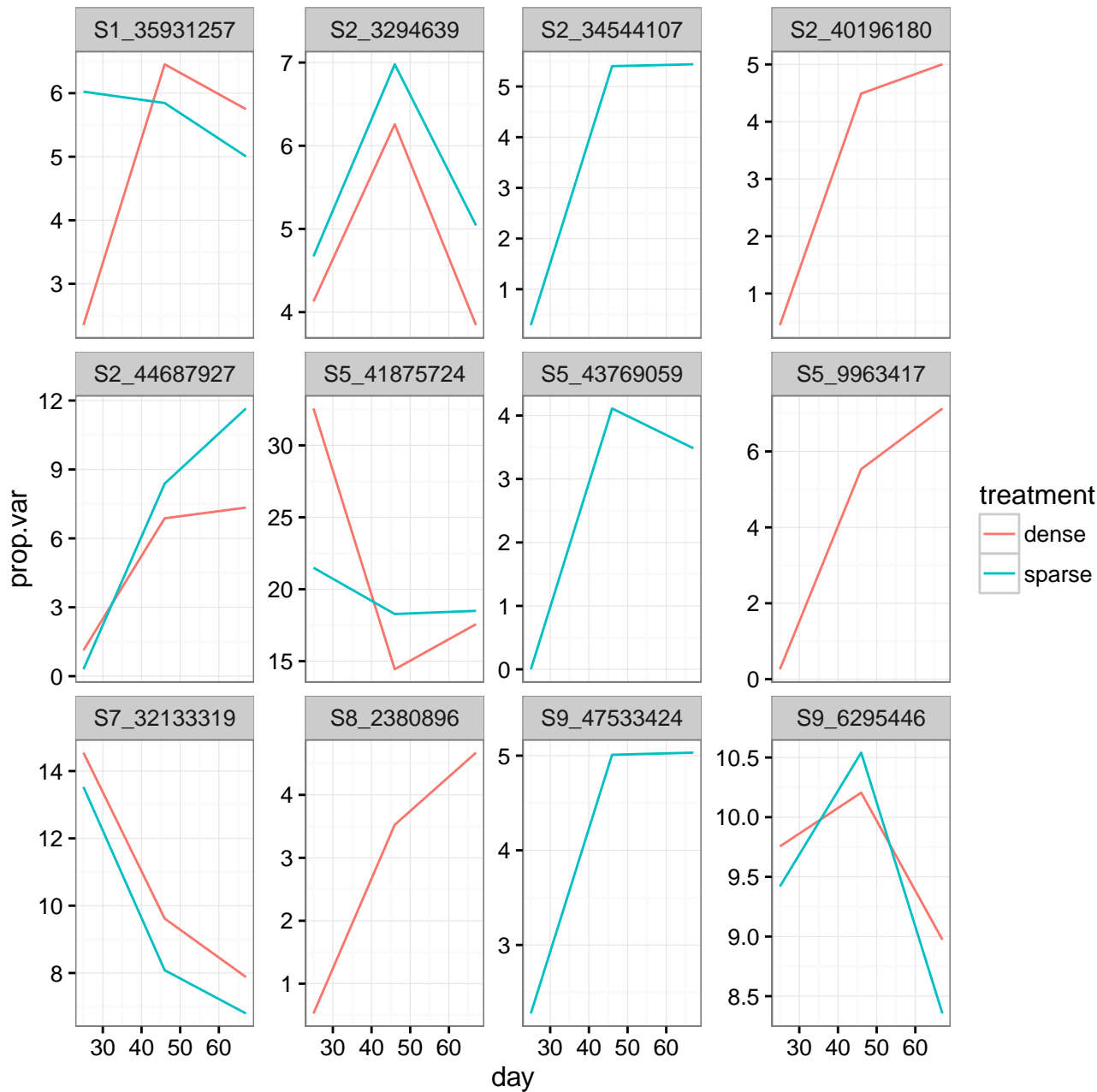

# DR13

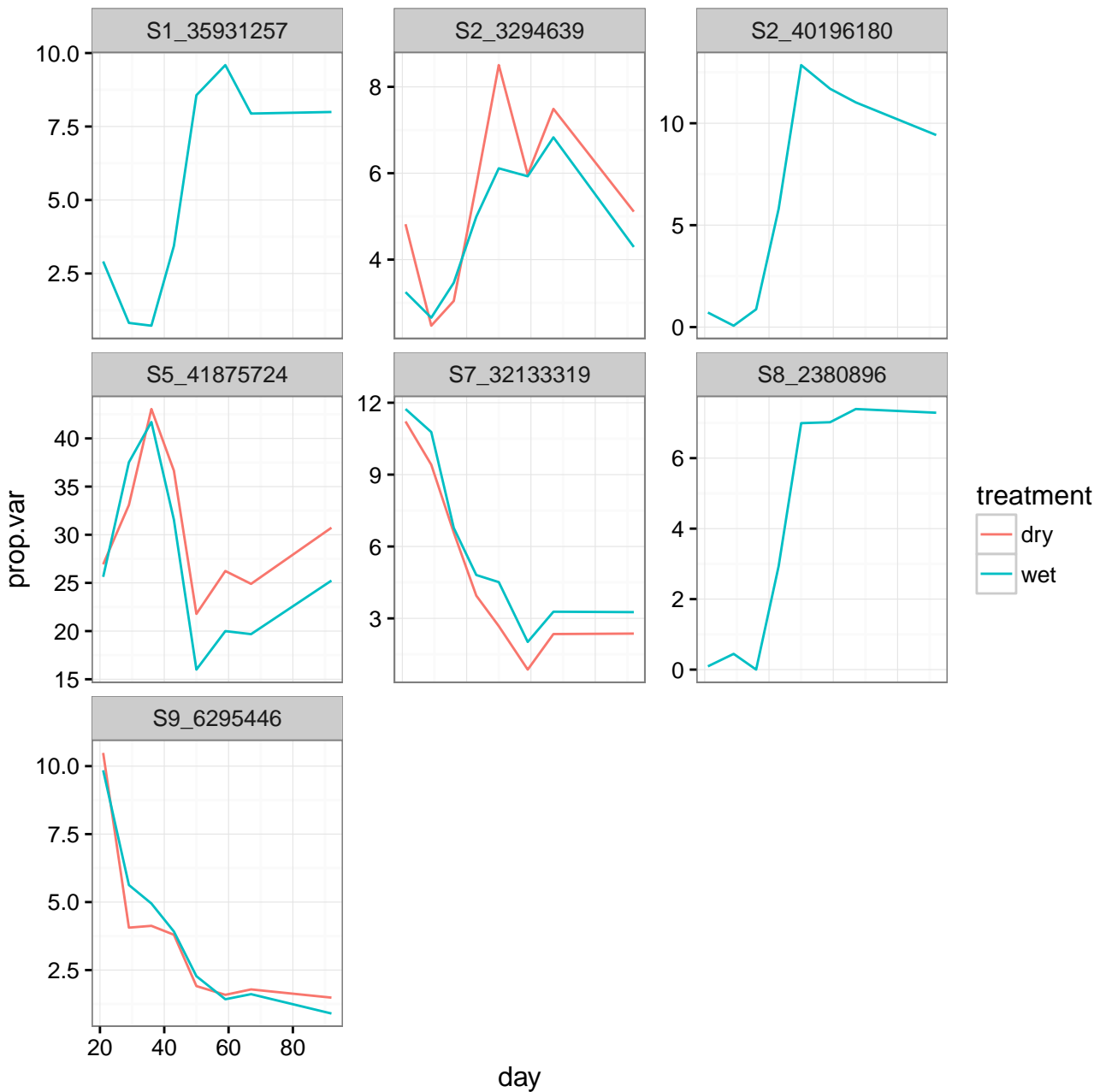

## BP14

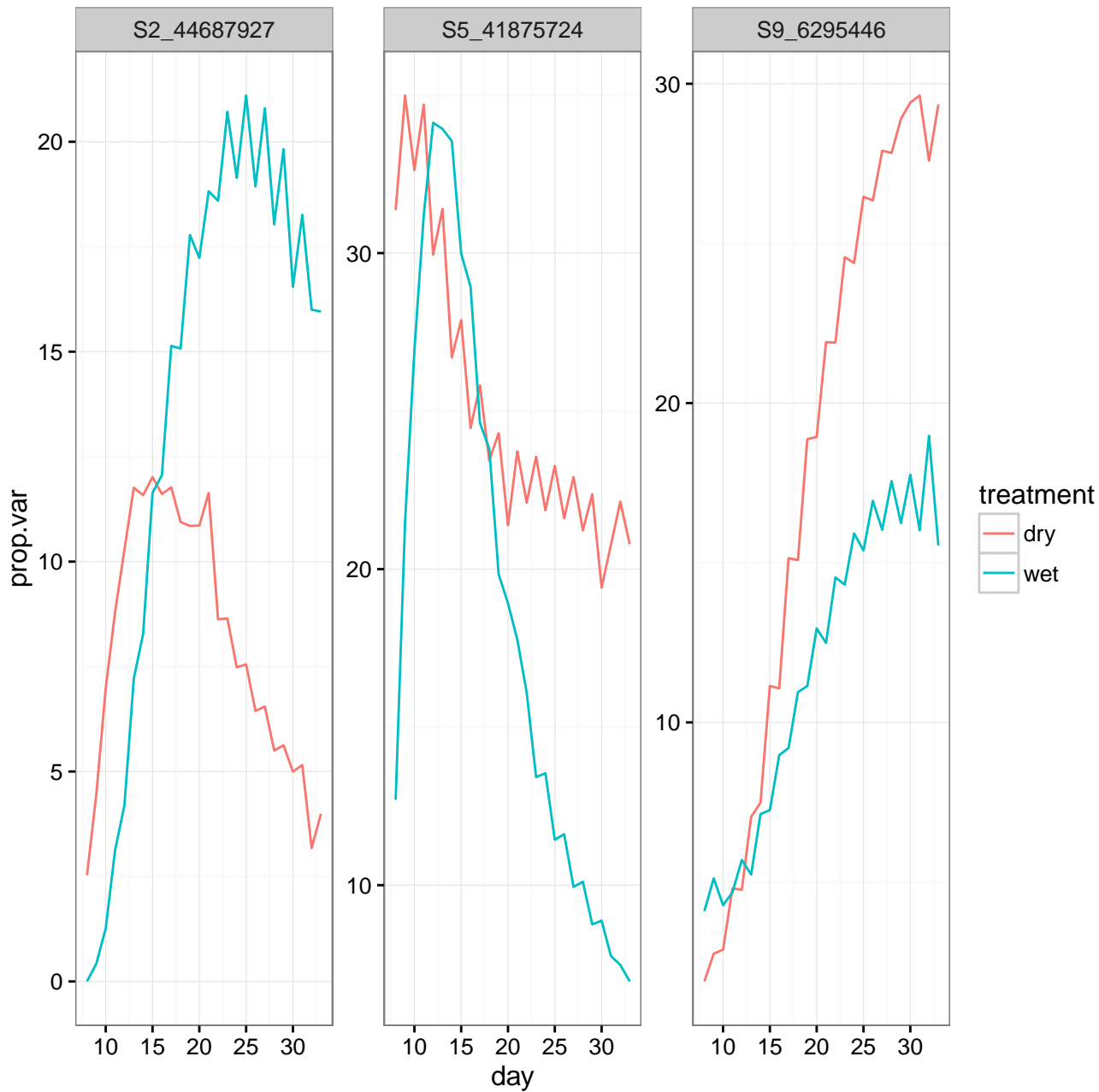

# DR14

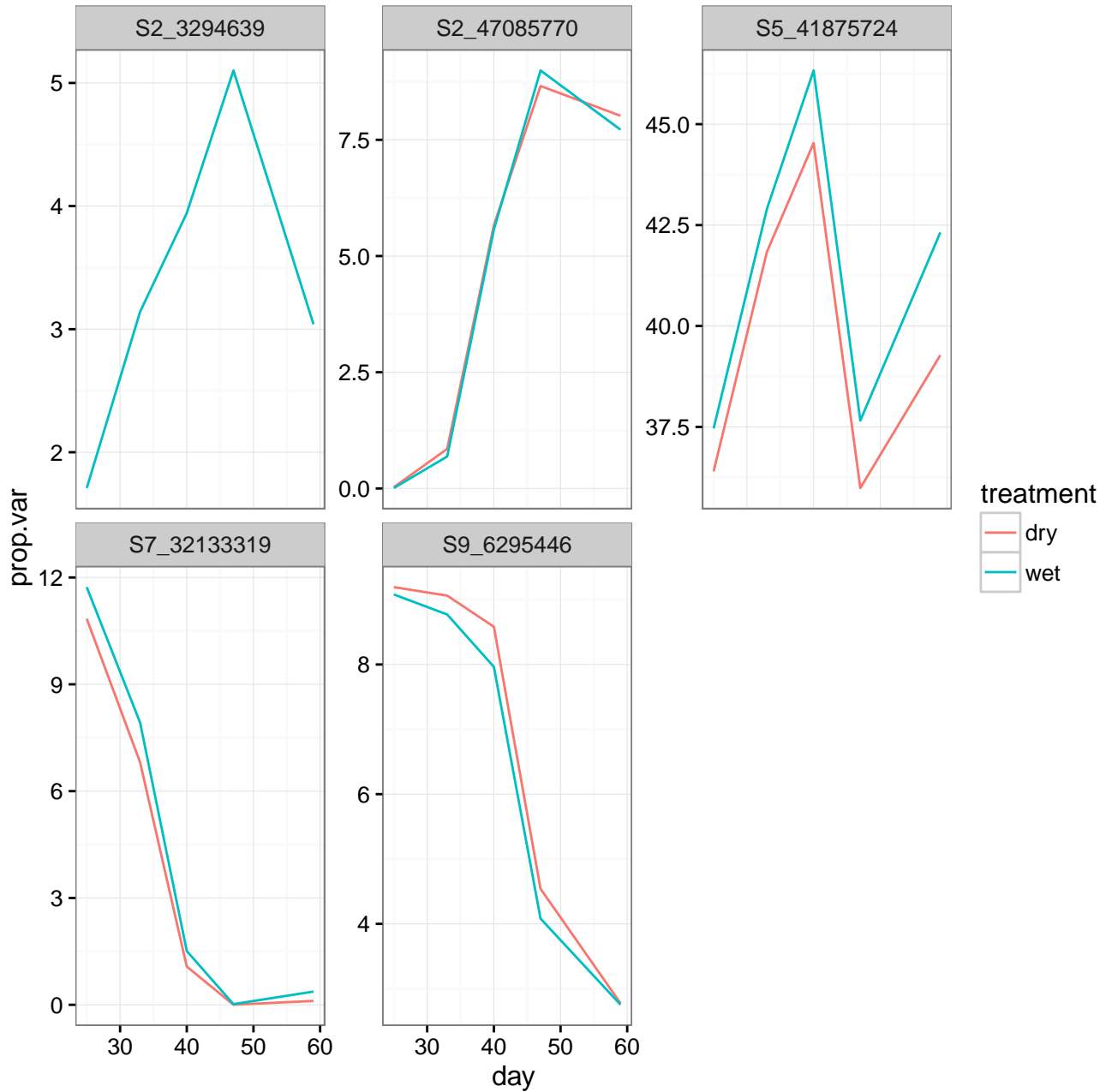

Supplement: S10 Fig — (PDF) [file pgen.1006841.s010.pdf]

# DN13

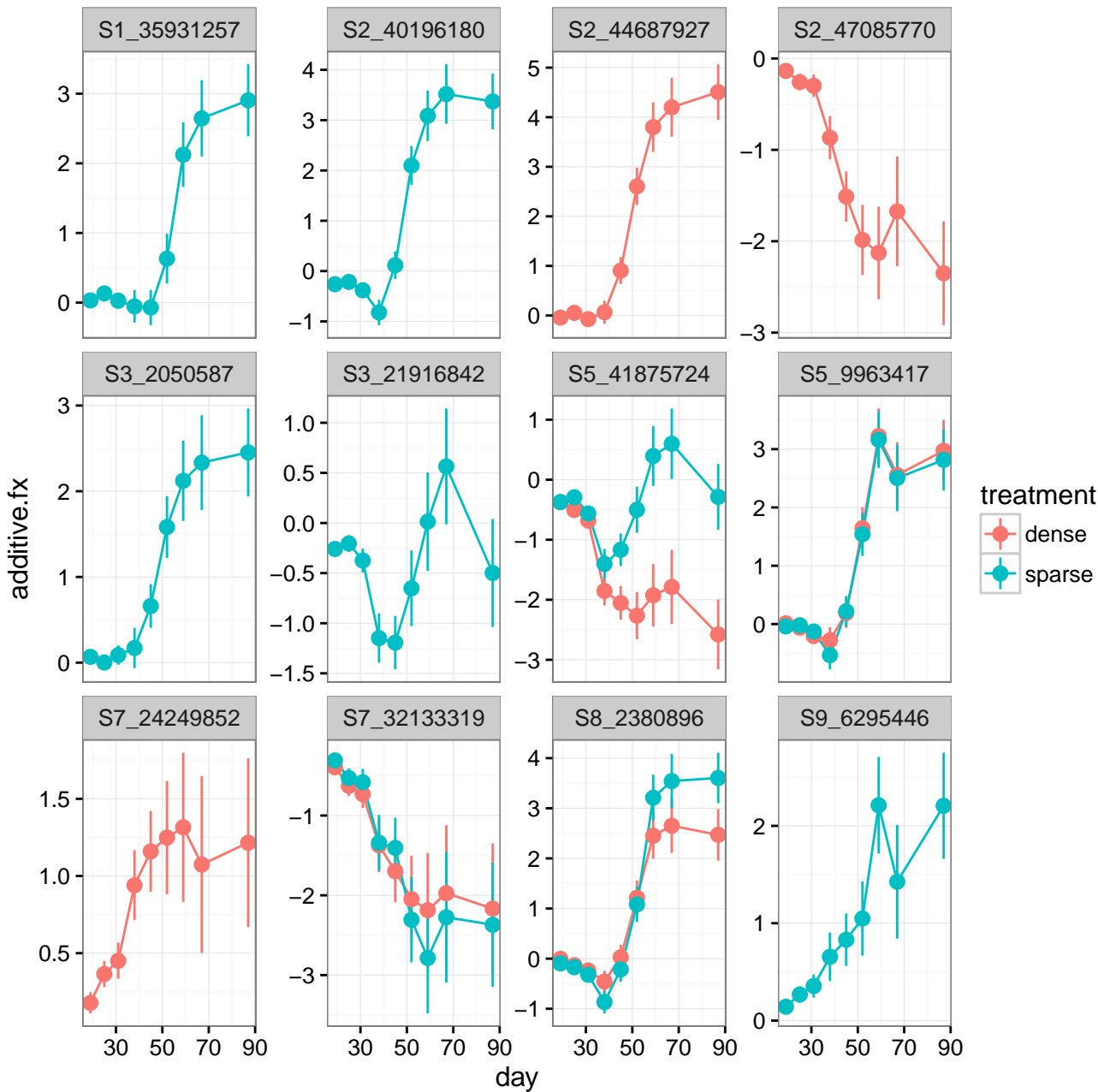

# DN14

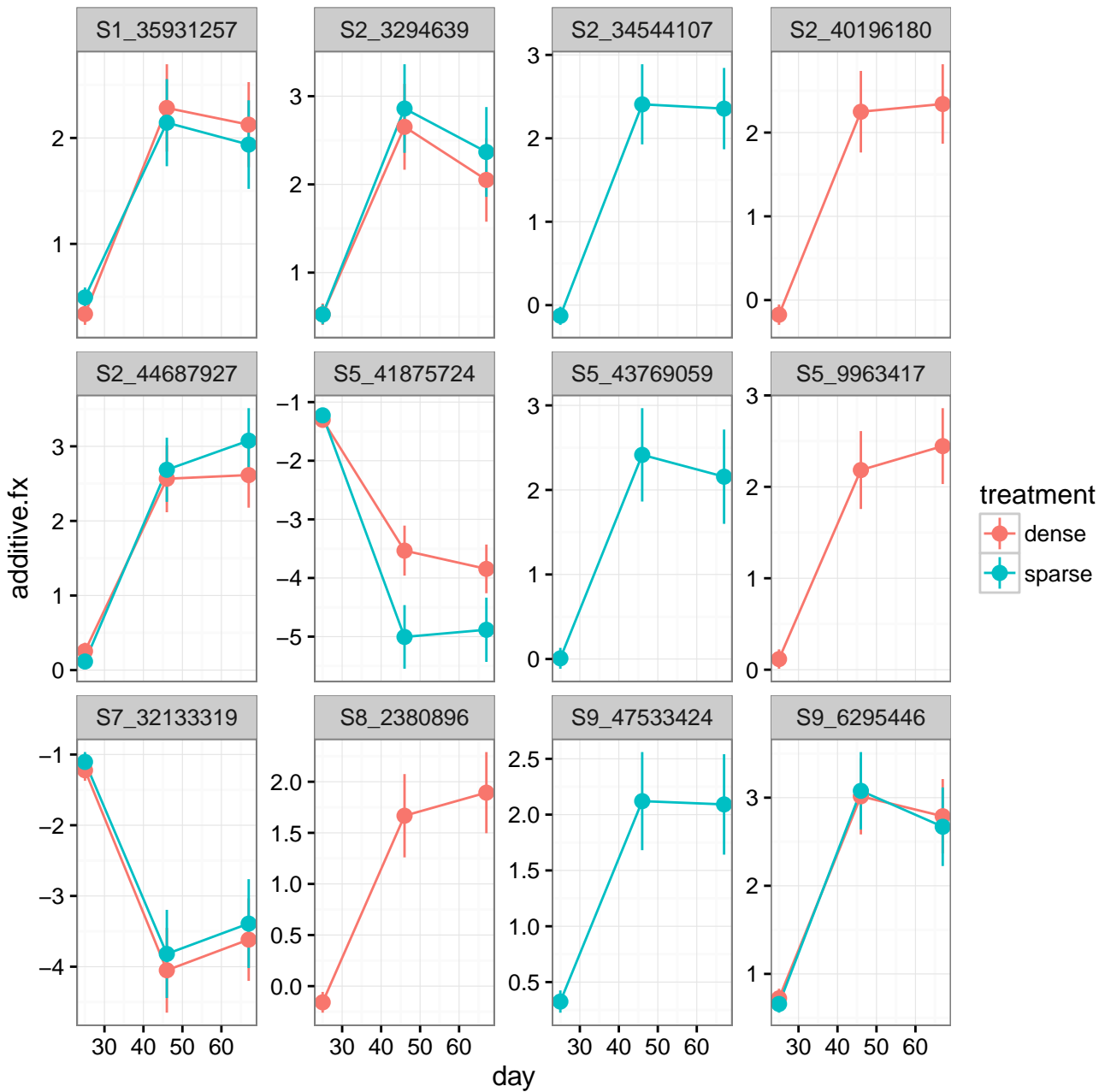

# DR13

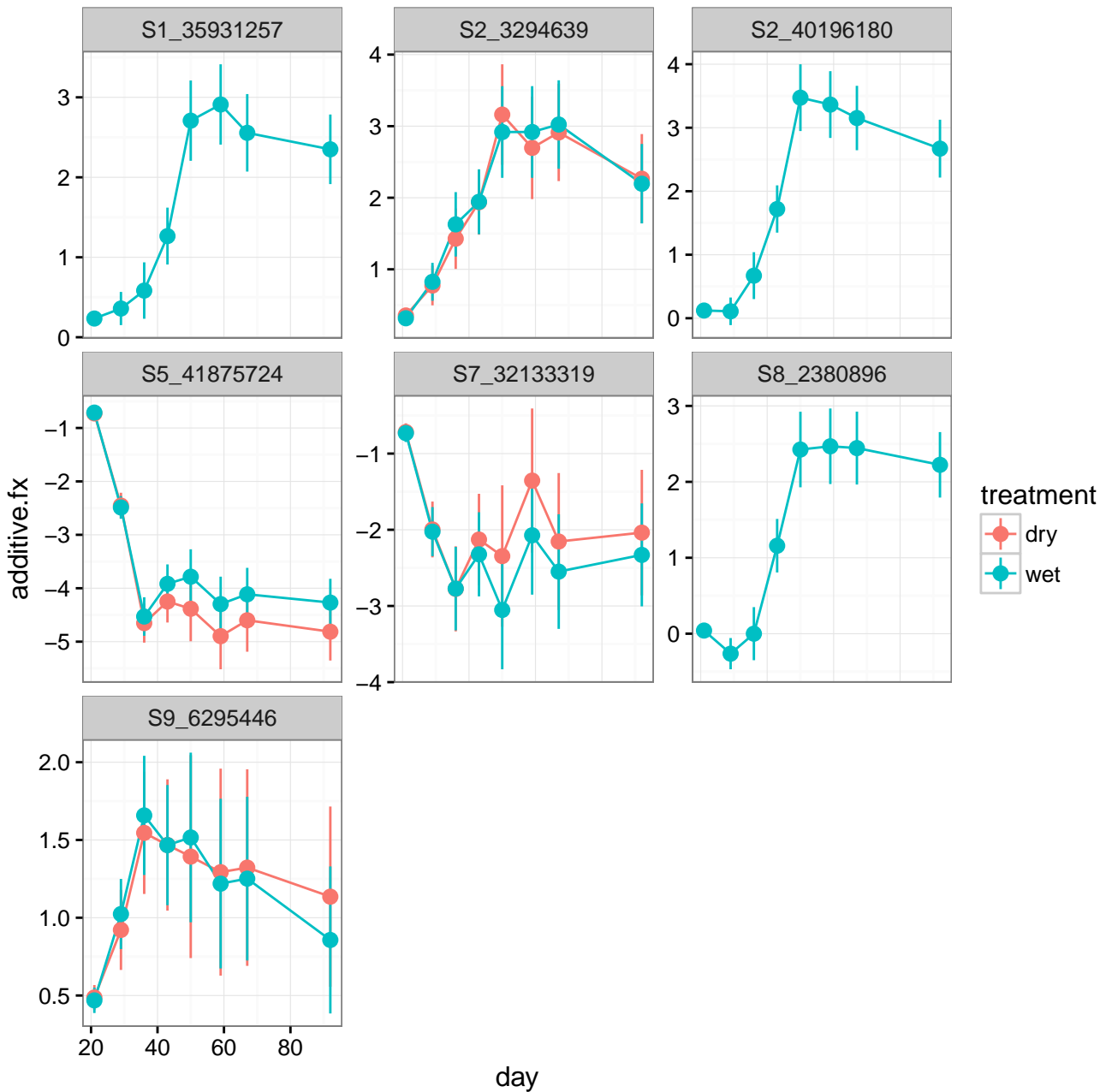

## BP14

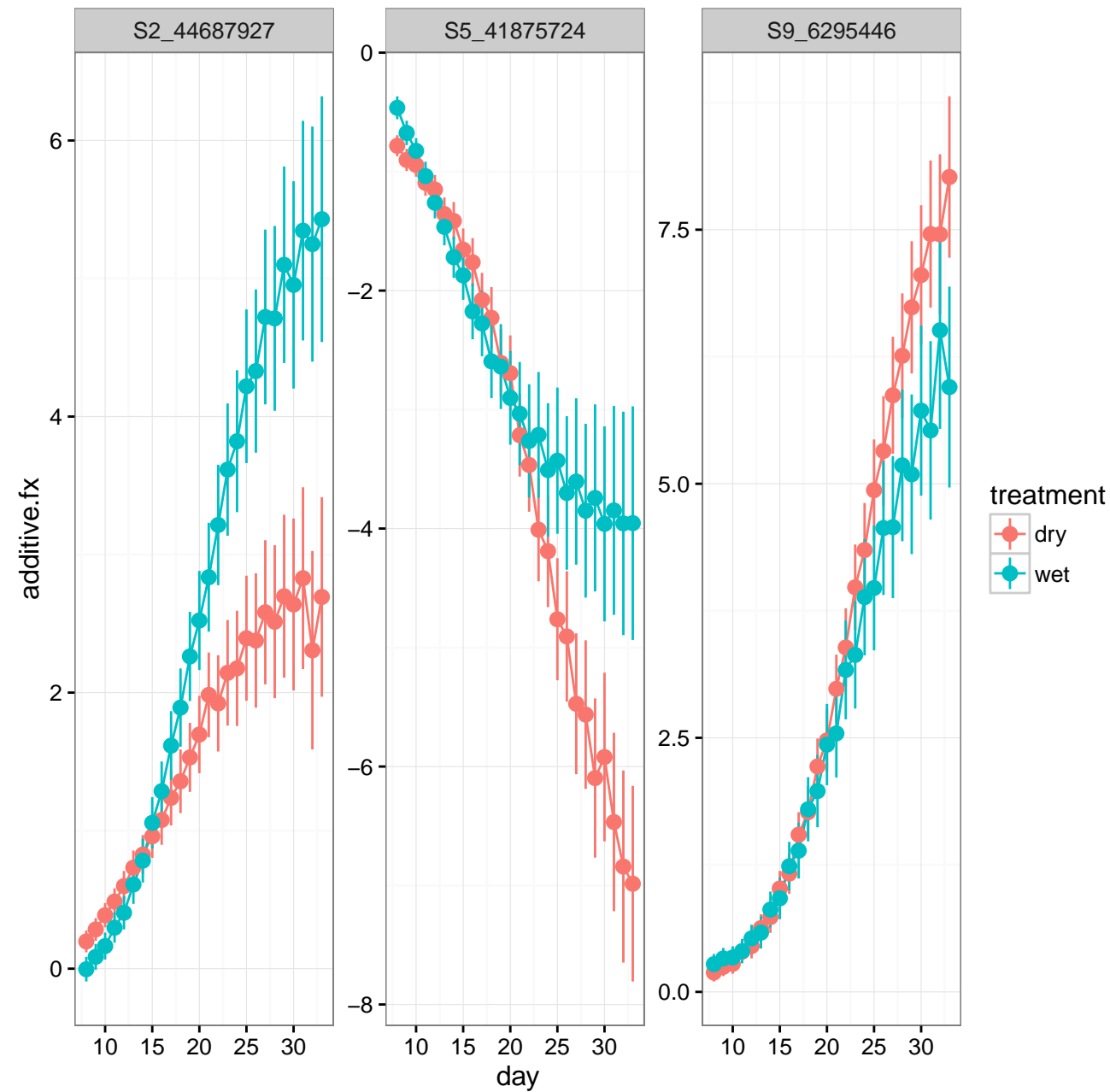

# DR14

additive.fx

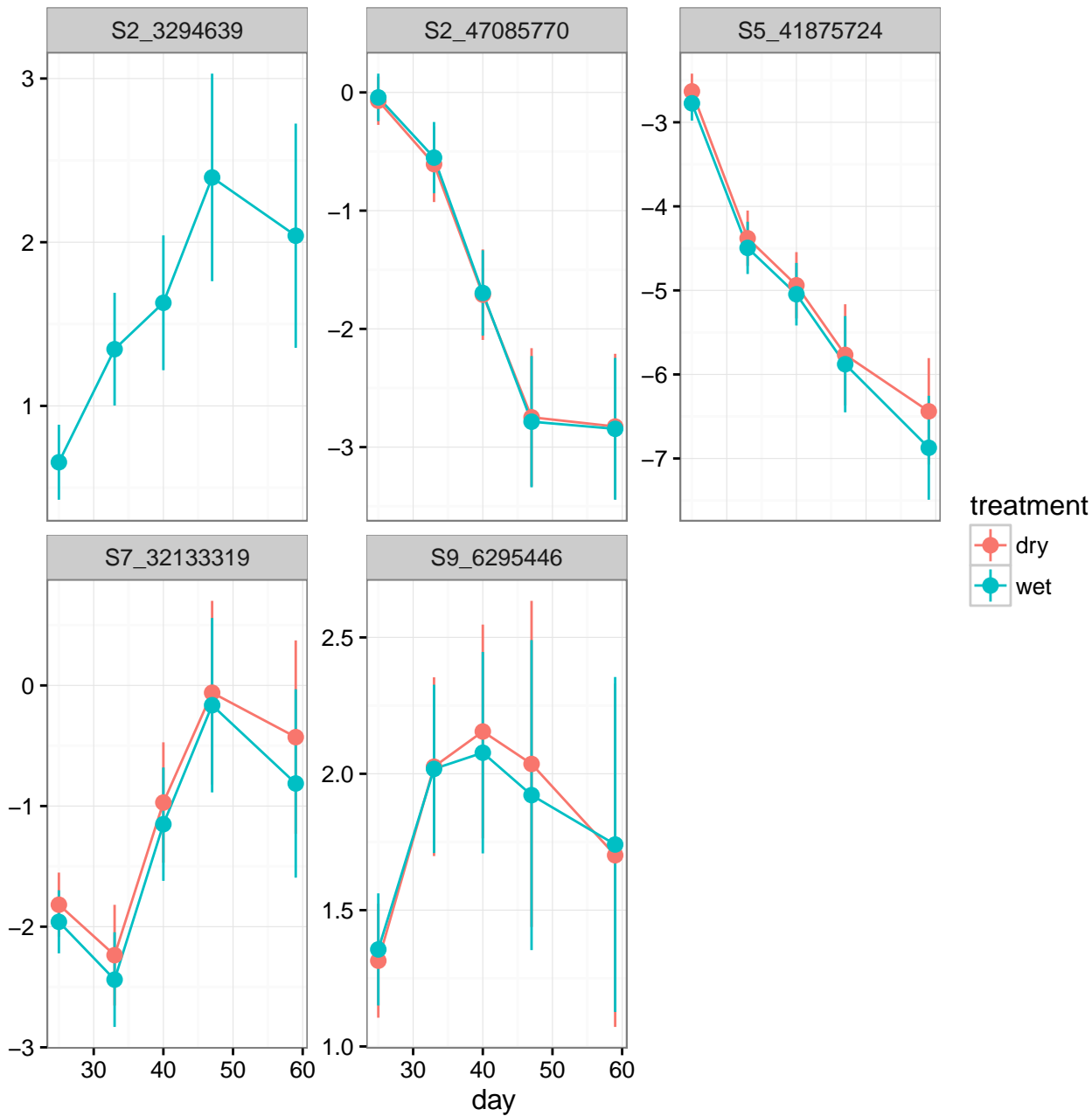

Supplement: S11 Fig — (PDF) [file pgen.1006841.s011.pdf]

S12a\_Fig

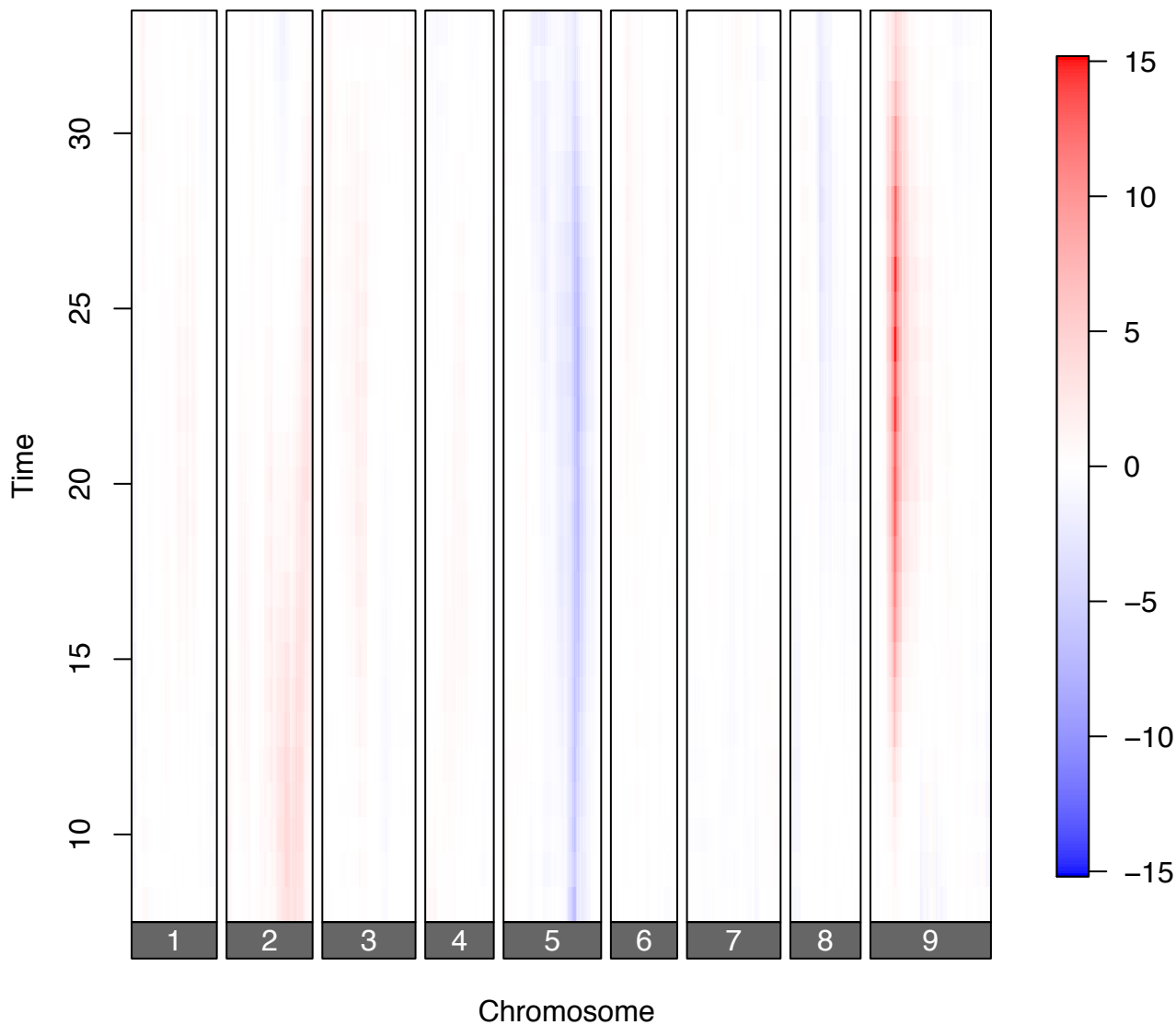

S12b\_Fig

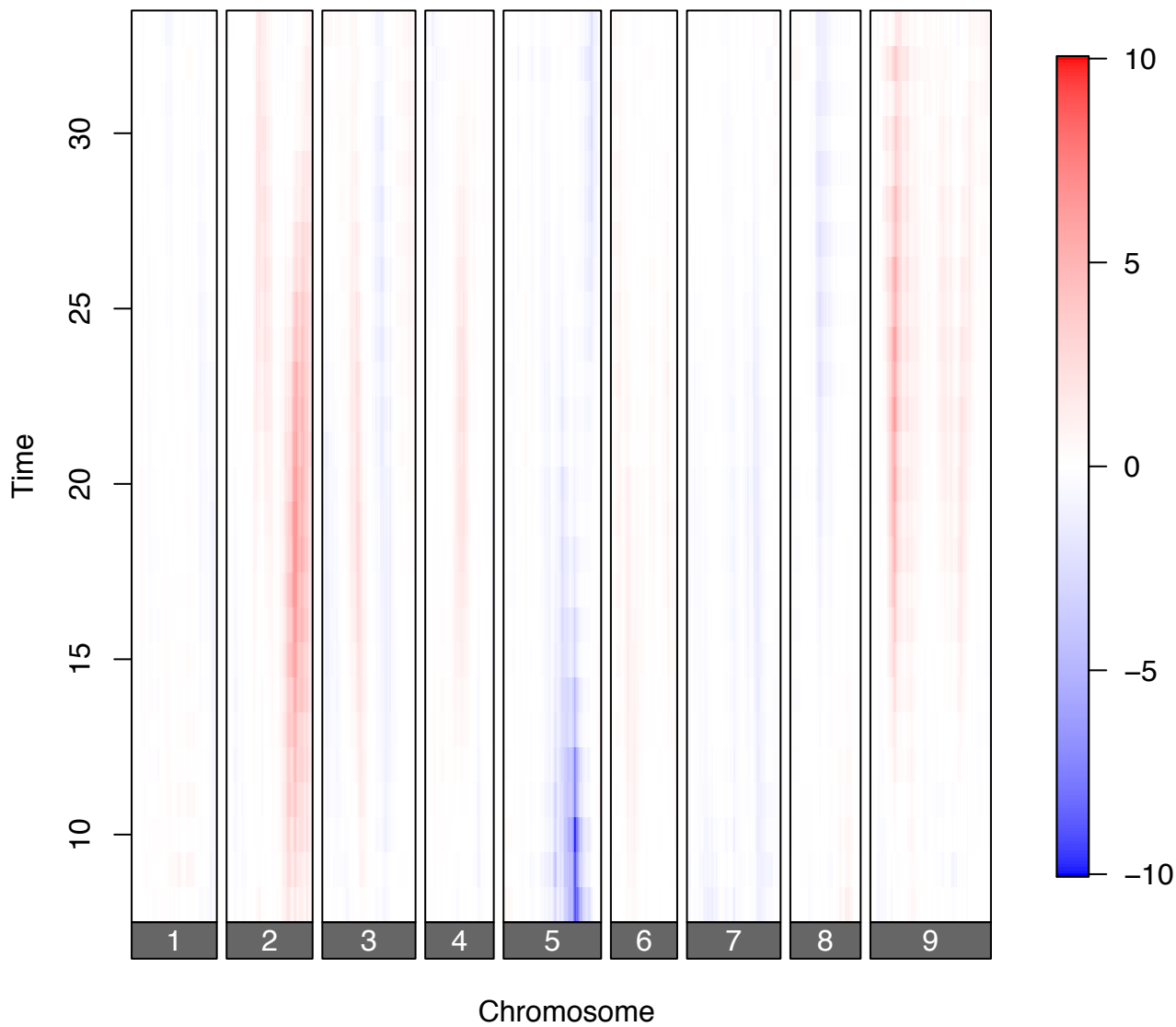

Supplement: S12 Fig — Loci at which the A10 allele contributes larger plant height are plotted in blue, whereas loci at which the B100 allele contributes to larger plant height are plotted in red. A) Water limited environment (Dry). B) Well-watered environment (Wet). (PDF) [file pgen.1006841.s012.pdf]

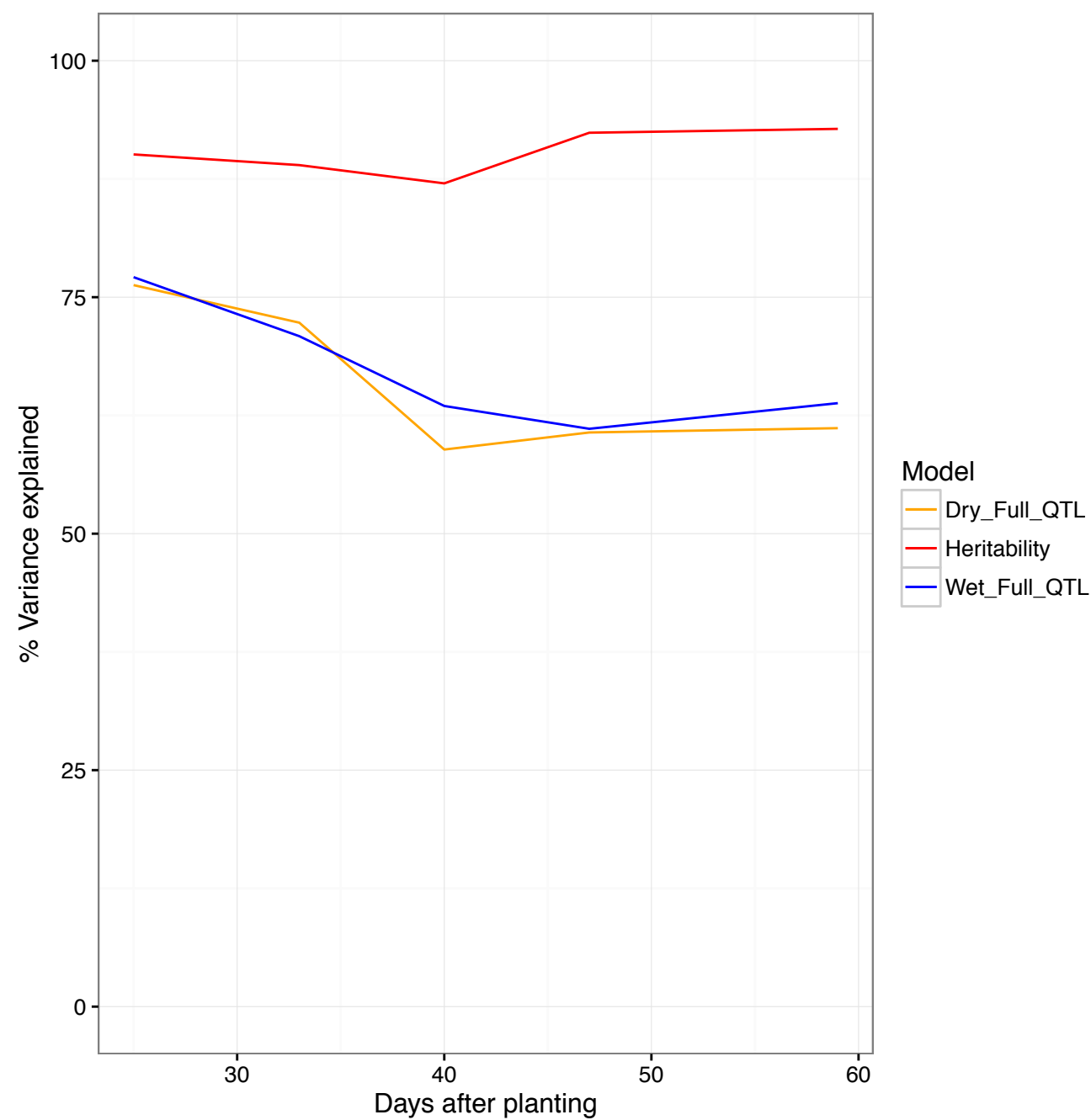

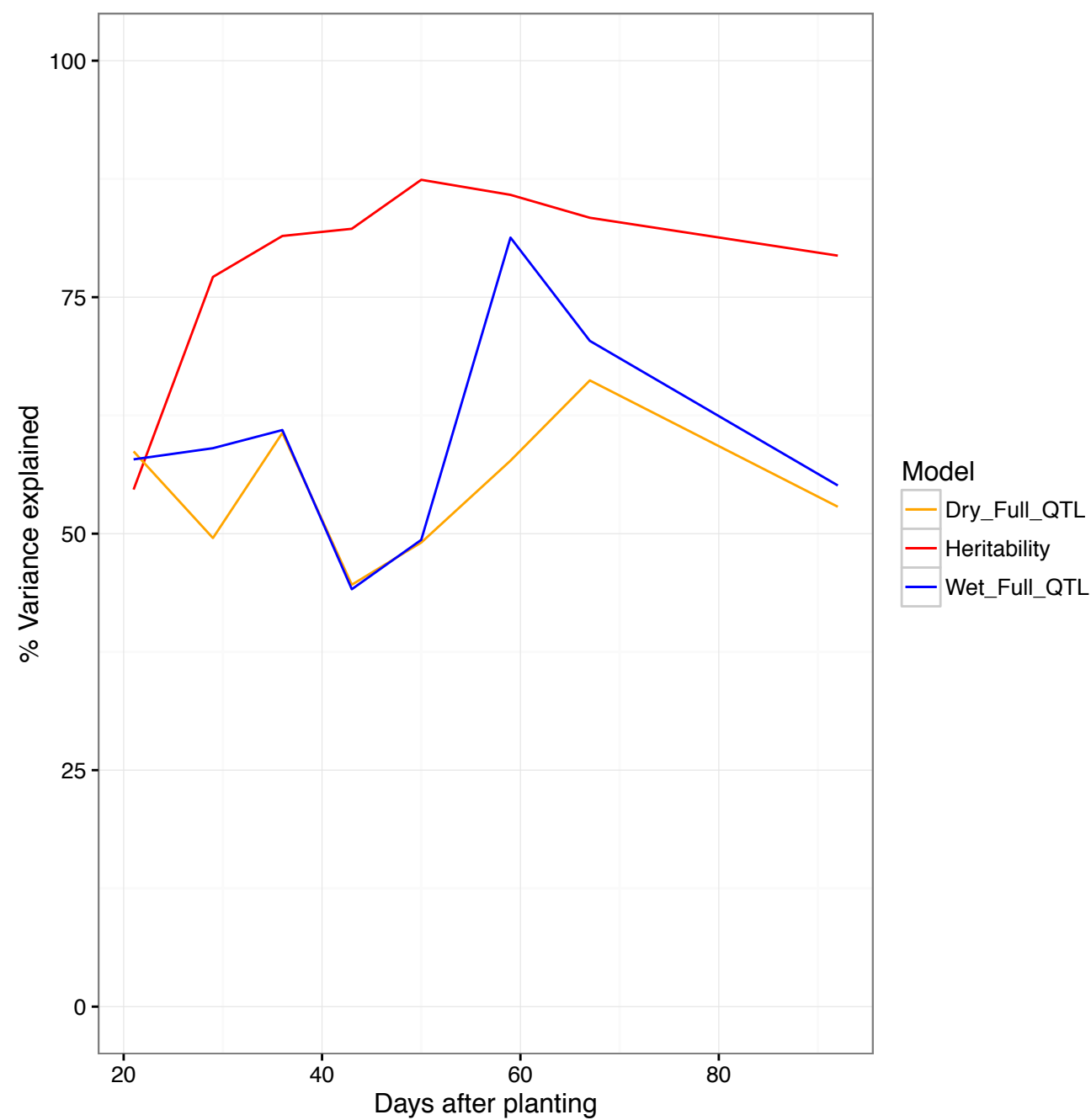

Supplement: S13 Fig — The discrepancy is particularly evident during the drought experiment performed in 2014 and in the dry treatment block in the 2013 drought experiment. A) The results of the drought experiment in 2014 B) The results of the drought experiment in 2013. (PDF) [file pgen.1006841.s013.pdf]

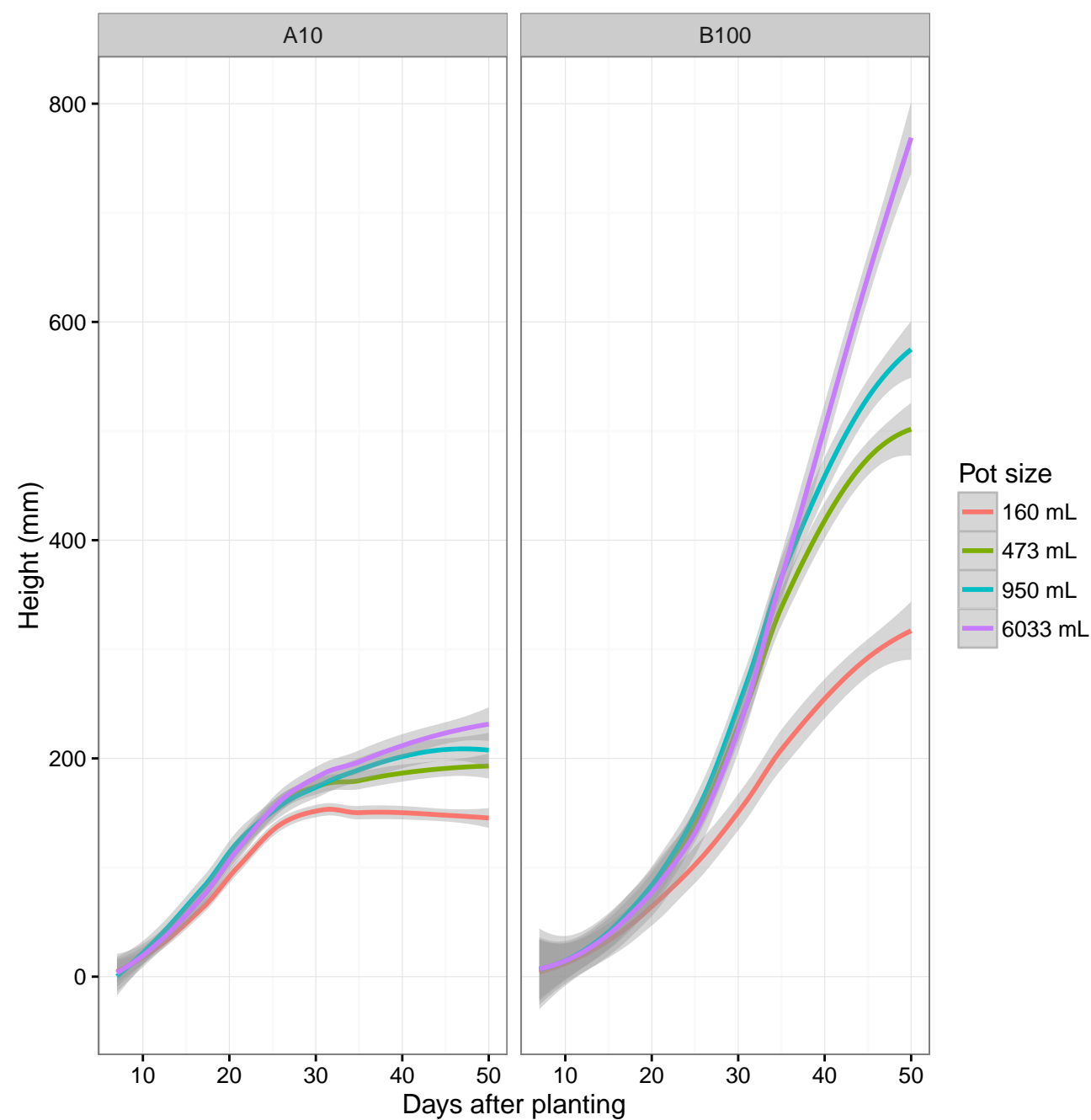

Supplement: S14 Fig — (PDF) [file pgen.1006841.s014.pdf]
