# Supplementary material for: Time dependent genetic analysis links field and controlled environment phenotypes in the model C4 grass Setaria
Source: PLoS Genet. 2017 Jun 23;13(6):e1006841. doi: 10.1371/journal.pgen.1006841 (PMC5507400; doi:10.1371/journal.pgen.1006841)

S7a_Fig

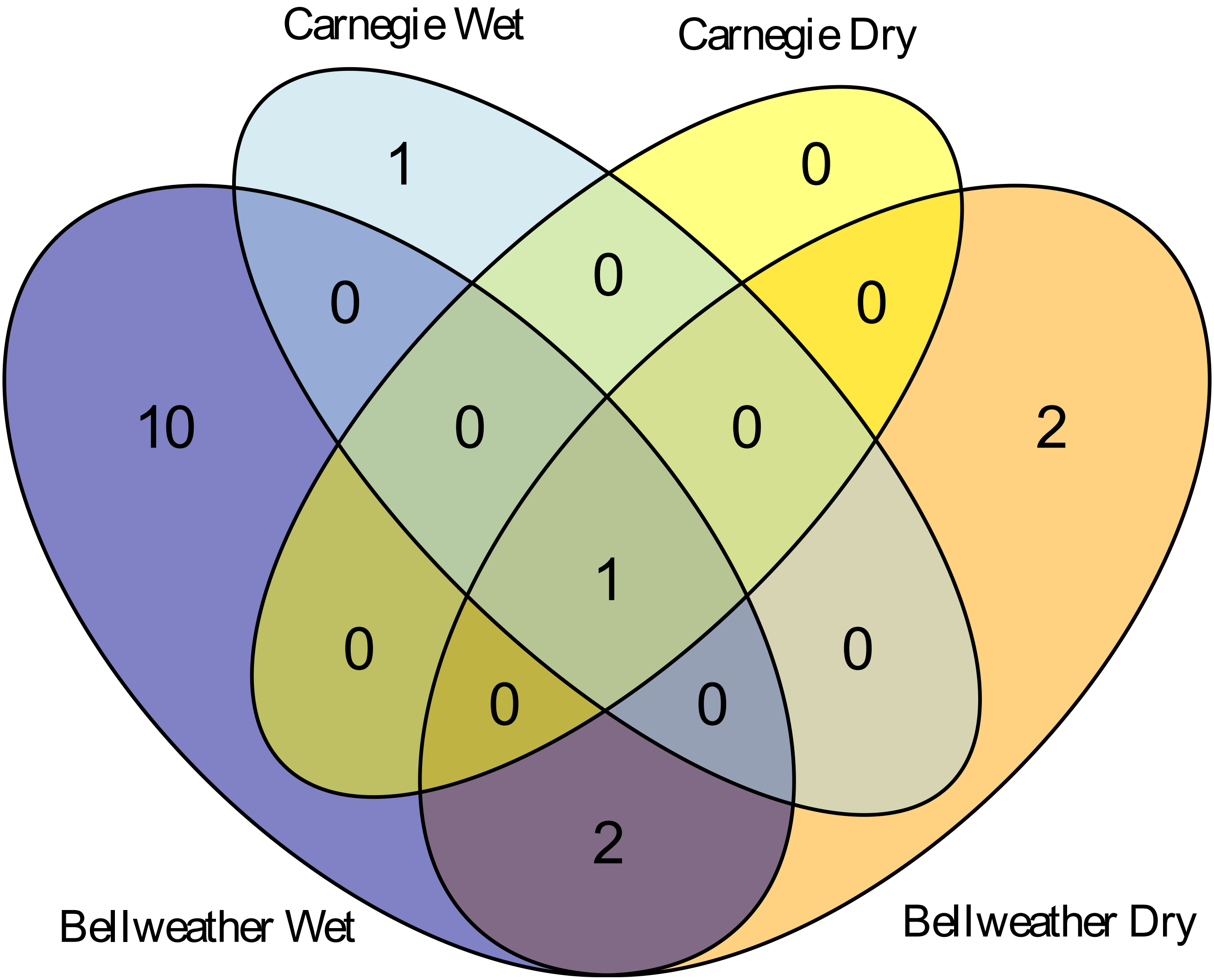

Carnegie Wet
Carnegie Dry
Bellweather Wet
Bellweather Dry
1
0
0
0
10
0
0
2
1
0
0
0
0
0
2

S7b_Fig

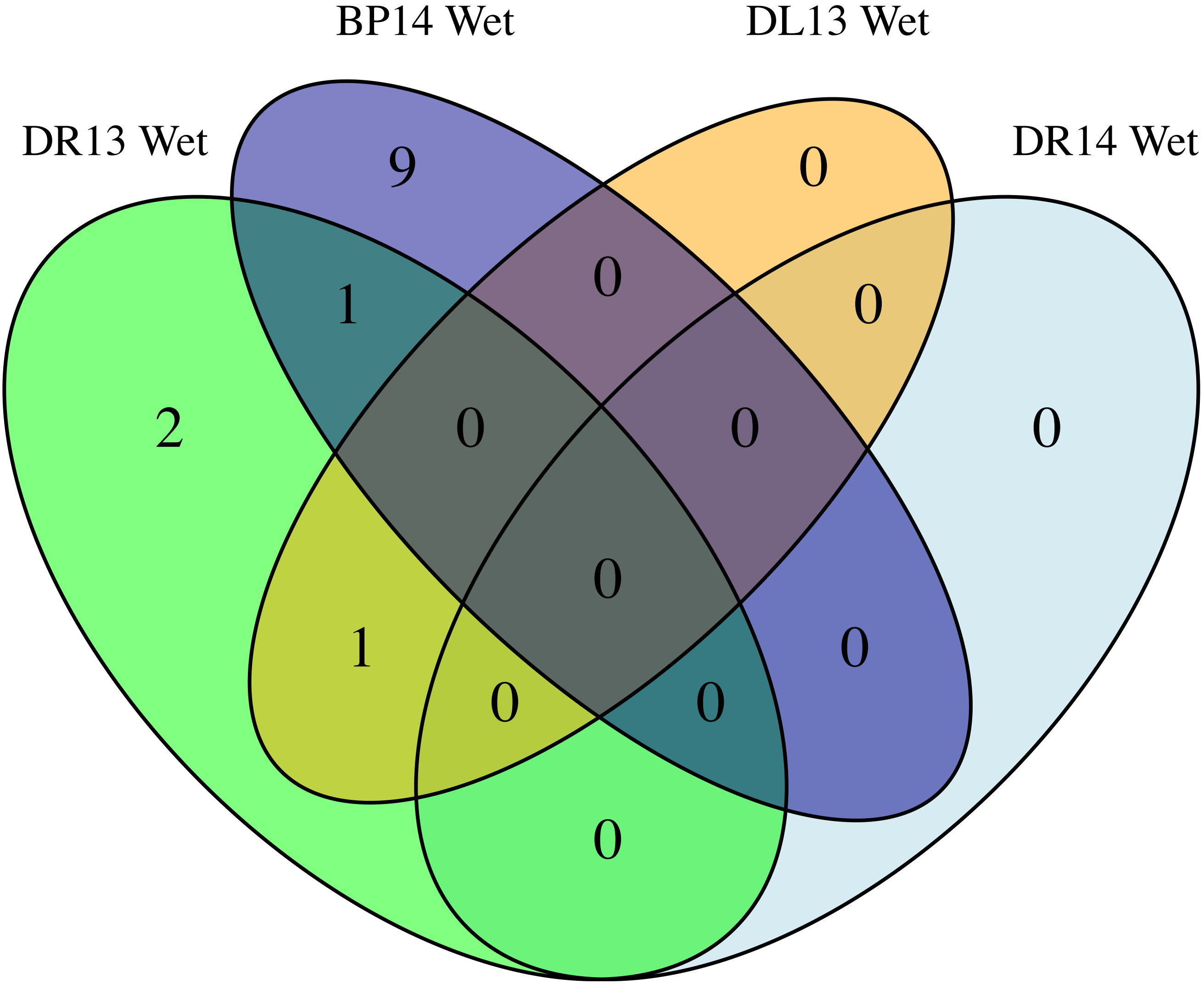

S7c_Fig

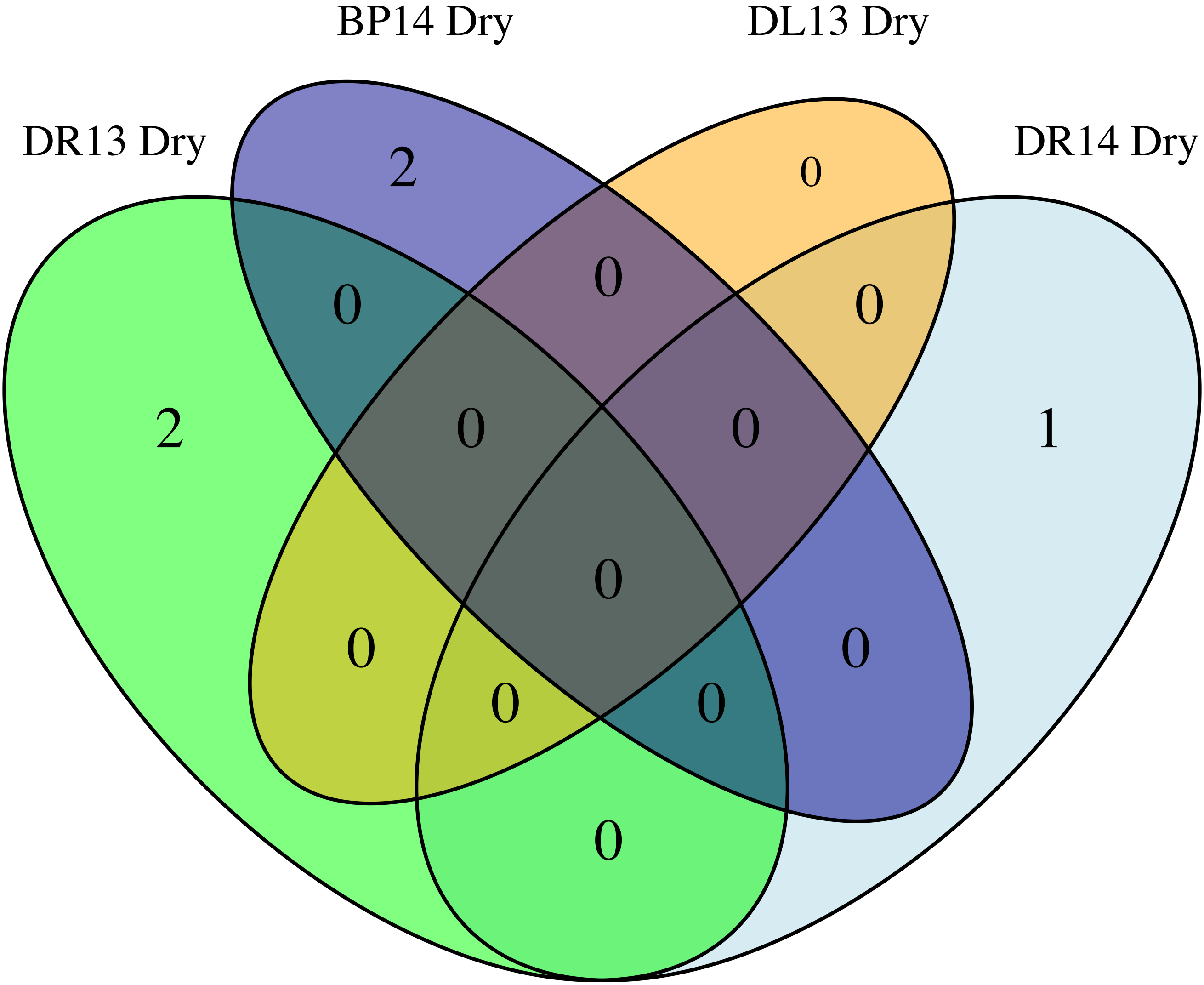

S7d_Fig

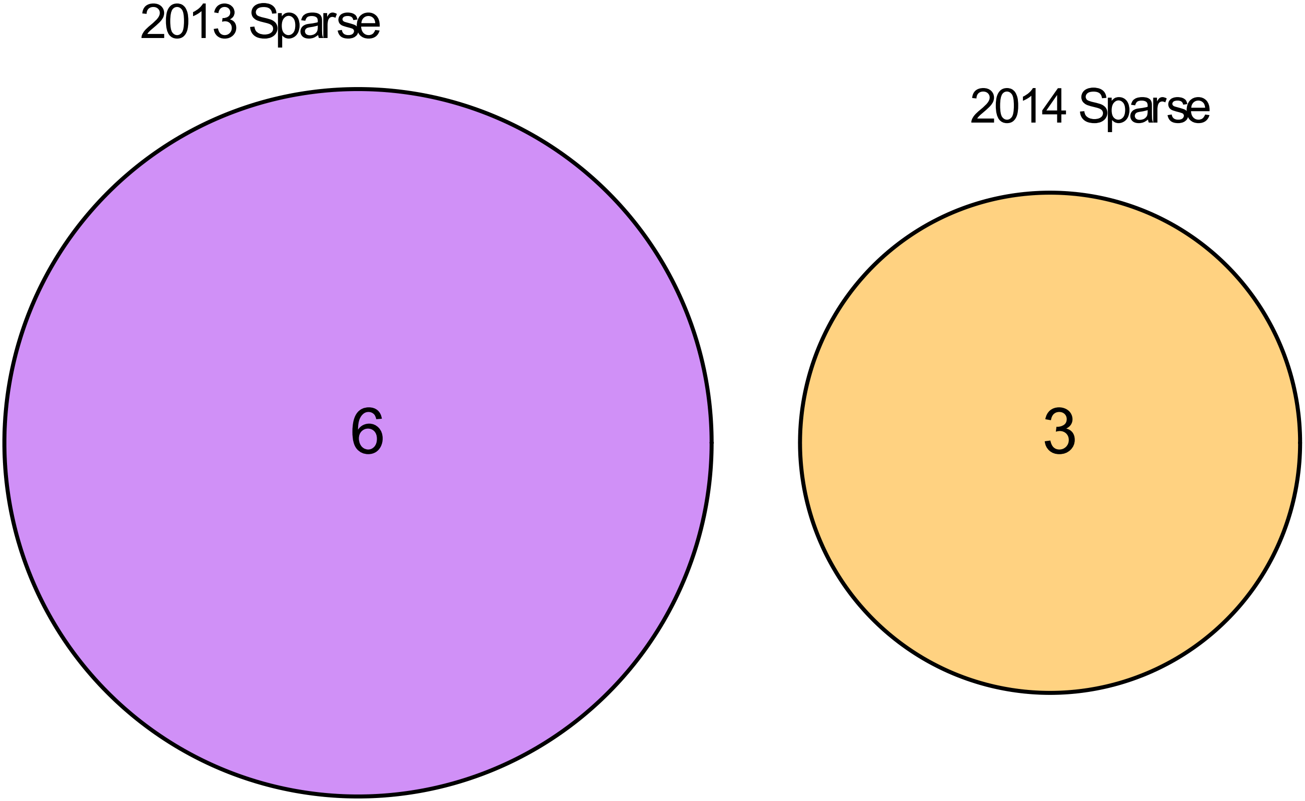

S7e_Fig

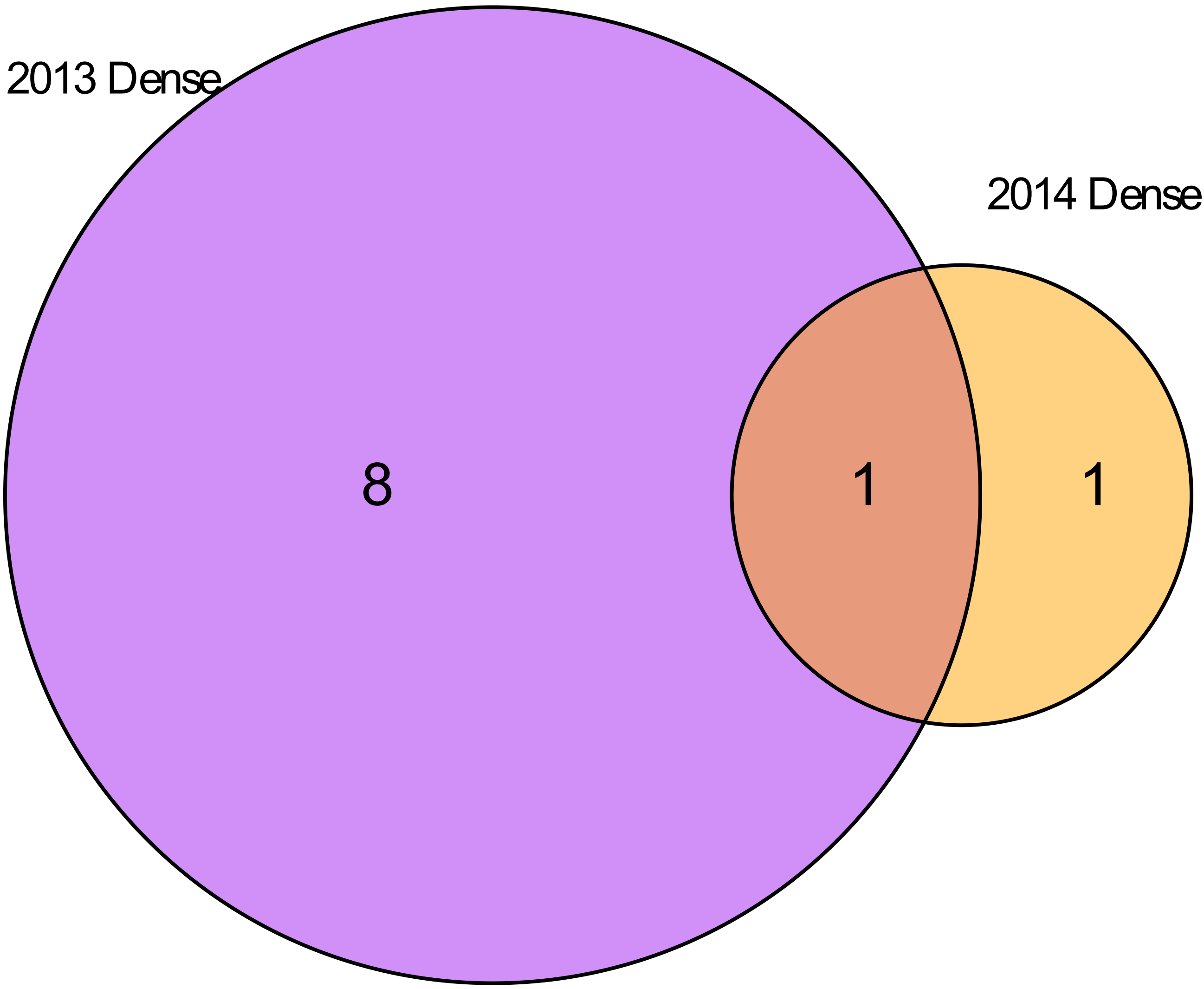

Supplement: S7 Fig — A) Overlap between QTL detected in treatment block and experimental locations within controlled environmental studies (Bellweather at Donald Danforth Plant Science Center compared to the growth chamber experiment at Carnegie Institute for Science). B) Among QTL found exclusively in wet treatment blocks there is no overlap between all experiments. C) Among QTL found exclusively in dry treatment blocks there is no overlap between all experiments. D) Among QTL found exclusively in sparse treatment blocks there is no overlap between all experiments. E) Among QTL found exclusively in dense treatment blocks there is one QTL found in both 2013 and 2014. (PDF) [file pgen.1006841.s007.pdf]
