# Supplementary material for: Time dependent genetic analysis links field and controlled environment phenotypes in the model C4 grass Setaria
Source: PLoS Genet. 2017 Jun 23;13(6):e1006841. doi: 10.1371/journal.pgen.1006841 (PMC5507400; doi:10.1371/journal.pgen.1006841)
Supplement: S9 Fig — (PDF) [file pgen.1006841.s009.pdf]

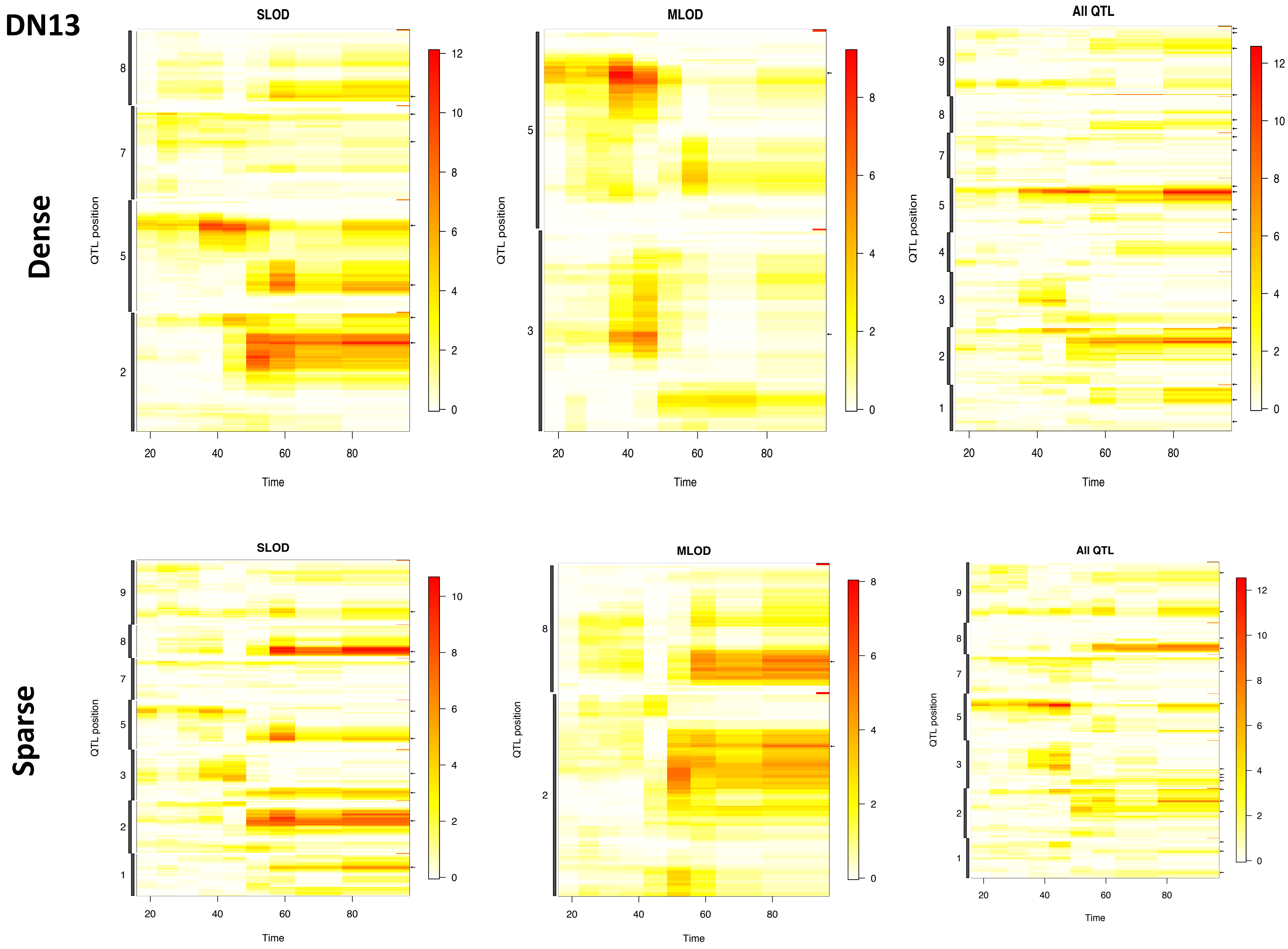
DN13
Dense
Sparse
SLOD
MLOD
All QTL
QTL position
Time
20
40
60
80
0
2
4
6
8
10
12

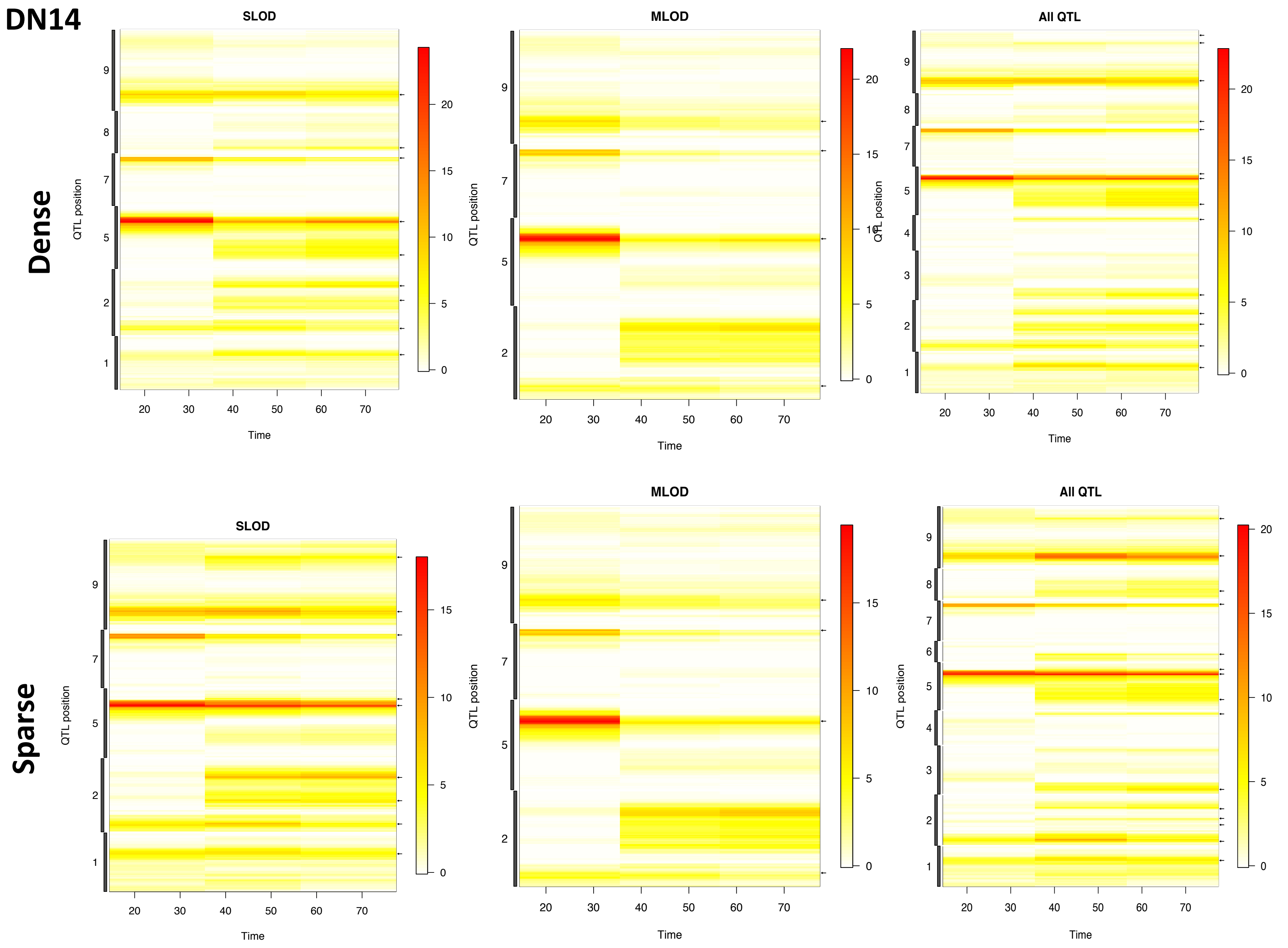

DN14
Dense
Sparse
SLOD
MLOD
All QTL
QTL position
Time

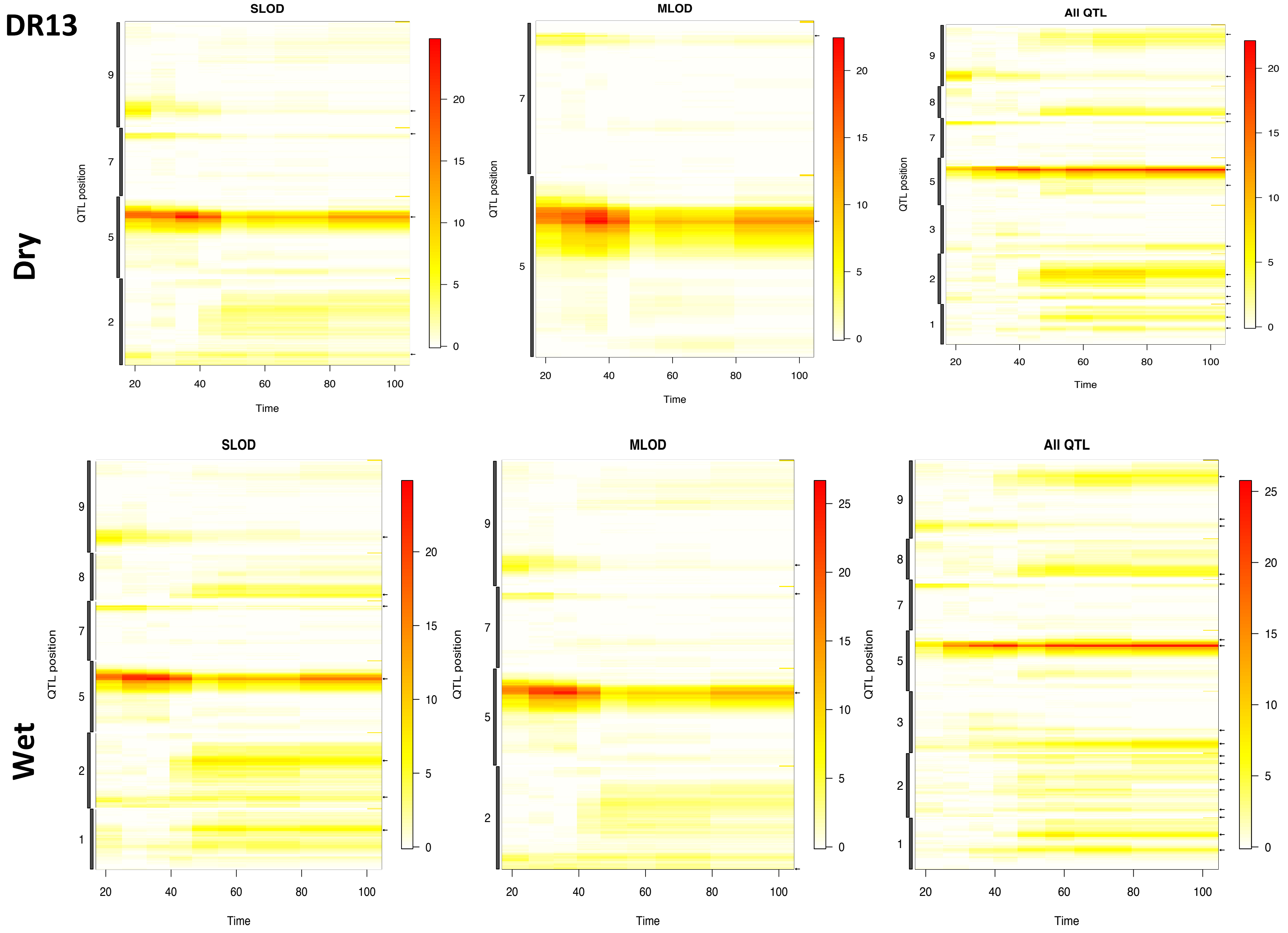
DR13
Dry
Wet
SLOD
MLOD
All QTL
QTL position
Time

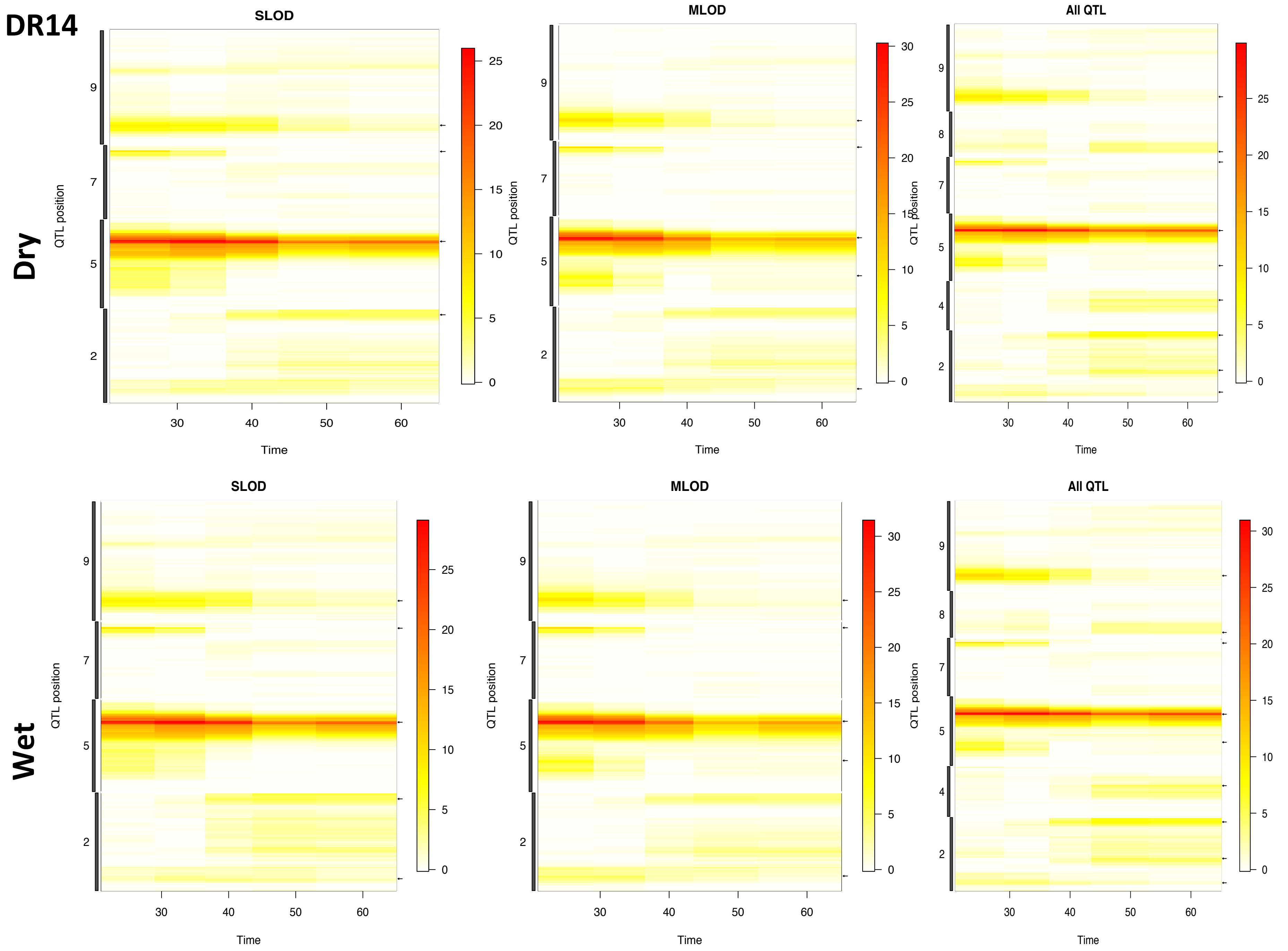
DR14
Dry
Wet
SLOD
MLOD
All QTL
QTL position
Time
9
8
7
5
4
2
30
40
50
60
0
5
10
15
20
25
30

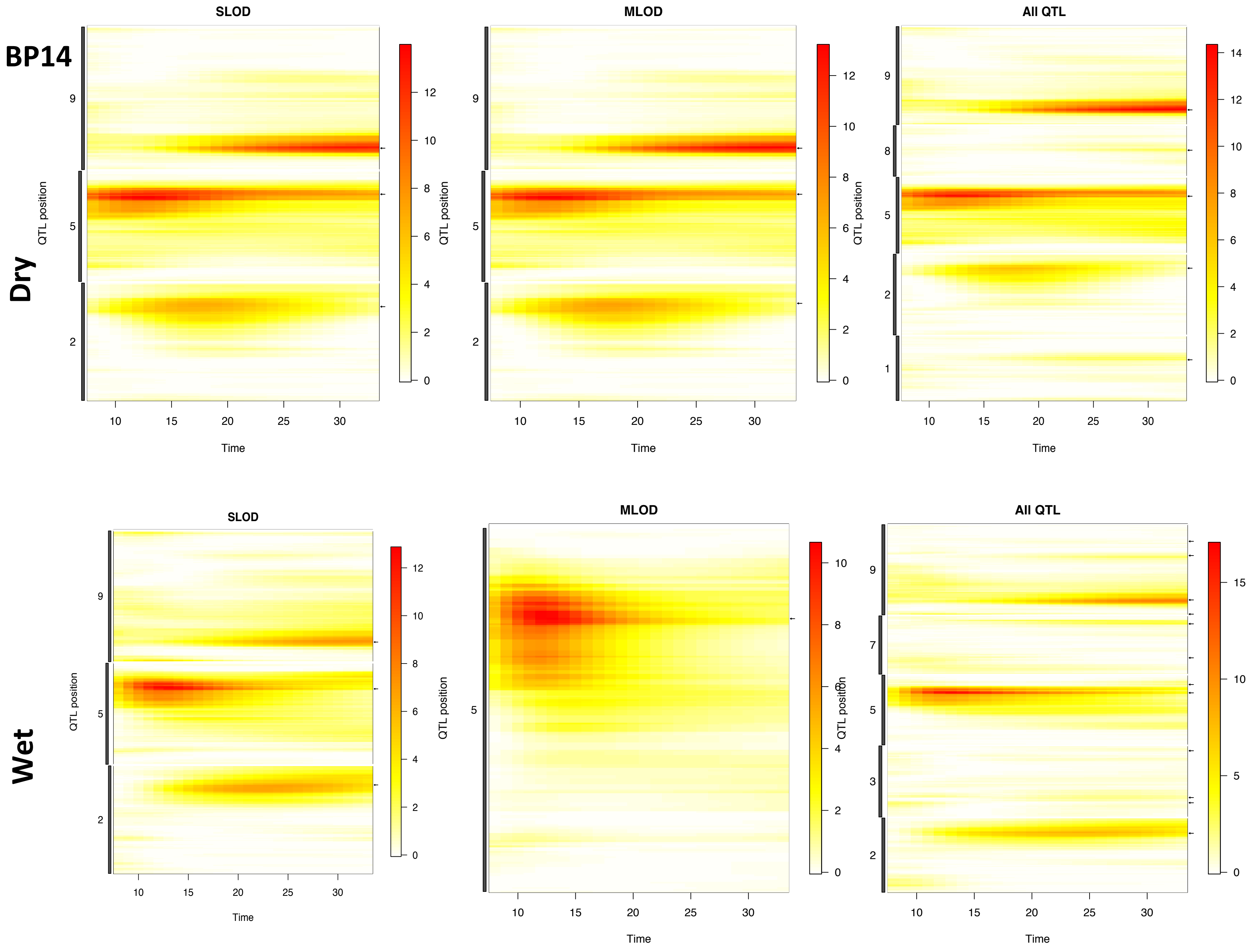
BP14
Dry
Wet
SLOD
MLOD
All QTL
QTL position
Time
